# Studies on the basic reproduction number in stochastic epidemic models with random perturbations

**DOI:** 10.1186/s13662-021-03445-2

**Published:** 2021-06-12

**Authors:** Andrés Ríos-Gutiérrez, Soledad Torres, Viswanathan Arunachalam

**Affiliations:** 1 Department of Statistics, Universidad Nacional de Colombia, Bogotá, Colombia; 2 CIMFAV, Universidad de Valparaiso, Valparaiso, Chile

**Keywords:** Basic reproduction number, Random perturbations, Brownian motion, Stability analysis

## Abstract

In this paper, we discuss the basic reproduction number of stochastic epidemic models with random perturbations. We define the basic reproduction number in epidemic models by using the integral of a function or survival function. We study the systems of stochastic differential equations for SIR, SIS, and SEIR models and their stability analysis. Some results on deterministic epidemic models are also obtained. We give the numerical conditions for which the disease-free equilibrium point is asymptotically stable.

## Introduction

Pandemics can cause sudden and drastic increases in mortality and morbidity rates as well as social, political, and economic disruptions. Humanity can defend itself against these types of problems with advances in science and with professionals in medicine, immunology, genetics, epidemiology, and statisticians. Finding the necessary measures to guarantee people’s access to medical centers is a topic of great interest; controlling the sources and vectors of contagion is the most efficient way to slow down a pandemic. Reducing infection rates guarantees not only well-being but also a reduction in mortality rates. Knowing the mechanisms of spread, infection, and death, modeling them mathematically, and making predictions of populations at risk are the most advantageous state tools to guarantee the right to life. Epidemic models are widely used to analyze the dynamics of populations under infectious diseases. They are crucial for studying the epidemic development and transmission dynamics of a disease. Mathematical models play an important role in predicting, assessing, and controlling potential outbreaks. One of the first epidemic models developed was the SIR model proposed in 1927 by Kermack and McKendrick (see [14]) based on the ordinary differential system given by equation (1.1). The SIR model is a compartmental model where the population is divided into different types of individuals: the susceptible ($S(t)$), the infected ($I(t)$), and the recovered ($R(t)$) individuals, respectively, at time *t*. The transmission-dynamic epidemic models help us understand that the risk of infection among susceptible individuals depends on the prevalence of infectious individuals. An infected individual becomes recovered after receiving treatment. We now give the system of differential equations:

1.1 $$\begin{aligned} \begin{aligned}& \frac{dS(t)}{dt}=-\beta I(t)S(t), \\ &\frac{dI(t)}{dt}=\beta I(t)S(t)-\gamma I(t), \\ &\frac{dR(t)}{dt}=\gamma I(t),\end{aligned} \end{aligned}$$

where *β* represents the rate of infection, the infection recovery rate is *γ*, and *N* is the total population size such that $S(t)+I(t)+R(t)=N\text{ for all }t$. However, these previous models do not assume the possibility of immigrants and emigrants. We consider a model with demography, for which *μ* is considered as the *emigration rate* and *η* is the *immigration rate*. Sometimes the rate *μ* is considered as the *mortality rate* and *η* is the *birth rate* in standard branching processes.

1.2 $$\begin{aligned} \begin{aligned} & \frac{dS(t)}{dt}=\eta N-\beta I(t)S(t)-\mu S ( t ), \\ &\frac{dI(t)}{dt}=\beta I(t)S(t)-\gamma I(t)-\mu I ( t ), \\ &\frac{dR(t)}{dt}=\gamma I(t)-\mu R ( t ).\end{aligned} \end{aligned}$$

We note that if $\eta = \mu $ then the population will be constant. In the above model, we assume that the disease for which infection does not confer immunity is called the *population of type SIS* (susceptible(S)–infection(I)–susceptible(S)) model since individuals return to the susceptible class when they recover from the infections. Such infections do not have a recovered state and individuals become susceptible again after recovery from infection. Now we describe the *population of type SEIR* (susceptible(S)–exposed(E)–infection(I)–recovered(R)), and the system of differential equations for the SEIR model (with demography) is given as follows:

1.3 $$\begin{aligned} \begin{aligned} &\frac{dS(t)}{dt}=\eta N-\beta I(t)S(t)- \mu S ( t ), \\ &\frac{dE(t)}{dt}=\beta I(t)S(t)-\upsilon E ( t ) -\mu E ( t ), \\ &\frac{dI(t)}{dt}=\upsilon E ( t ) -\gamma I(t)-\mu I ( t ), \\ &\frac{dR(t)}{dt}=\gamma I(t)-\mu R ( t ),\end{aligned} \end{aligned}$$

where the *average incubation time* $1/ \upsilon $ is the time for which the infectious agent takes a time to convert an *exposed* individual into an *infected* individual. Note that during *incubation time* the *exposed individual* cannot transmit the disease. The above models are *deterministic*. However, the epidemics tend to occur in cycles of outbreaks due to variations in the infection rate mainly related to certain external factors such as people’s social activities and climatic fluctuations (see [24]). In fact, the *climatic variations* can affect the *infection rate* $(\beta ) $. The epidemic models with random perturbation have been widely studied to accommodate randomness in the model, see for example [3, 7, 13, 20, 27]. More recently the evidence of the mechanism by which climate change could have played a direct role in the emergence of COVID-19 has been reported [2].

In this paper, we study the basic reproduction number in epidemic models with random perturbations. We define the basic reproduction number in epidemic models by using the survival function and demonstrate the numerical conditions under which the disease-free equilibrium point is asymptotically stable. The paper is organized as follows: In Sect. 2, we introduce the framework and basic concepts of the stochastic models with random perturbation and establish the stability conditions of the SIS, SIR, and SEIR epidemic models. Section 3 is devoted to the main results illustrated with simulation results for the basic reproduction number for the SIR, SIS, and SEIR models. Section 4 discusses the basic reproduction variable with double perturbation terms for the transmission rate; and finally, Sect. 5 concludes the paper with the future work.

## Stochastic model

In this section, we introduce the stochastic modeling of epidemics with *random perturbations*. In our model, we consider environmental variations and social behaviors in the infection rate [9]. In this paper, we assume $(\Omega, \Im,, \{\Im _{t}\}_{t\geq 0} \mathbb{P}, )$ to be a complete probability space with a filtration $\{\Im _{t}\}_{t\geq 0}$ satisfying the usual conditions. We define

2.1 $$\begin{aligned} \widetilde{\beta }:=\beta +\sigma B ( t ), \end{aligned}$$

where *β* and *σ* are positive constants, and $\{ B ( t ) \} _{t\geq 0}$ is the standard Brownian motion with $B ( T ) -B ( t ) \sim \mathbf{N} ( 0,T-t ) $. We note that the constant *β* is the deterministic mean *infection rate*, and *σ* is the *perturbation parameter* which describes changes in the infection rate changes over time with respect to *β*. We now introduce the stochastic perturbations (1.1) in the system of *stochastic differential equations(SDE)* for the SIR model. The resulting SDE is given by

2.2 $$\begin{aligned} \textstyle\begin{cases} dS(t)= ( \eta N-\beta I(t)S(t)-\mu S ( t ) ) \,dt-\sigma I(t)S(t)\,dB ( t ), \\ dI(t)= ( \beta I(t)S(t)-\gamma I(t)-\mu I ( t ) ) \,dt+\sigma I(t)S(t)\,dB ( t ), \\ dR(t)=\gamma I(t)-\mu R ( t ).\end{cases}\displaystyle \end{aligned}$$

Reasoning analogously as in (2.2), we now propose the following system of stochastic differential equations for the *SEIR model with random perturbations*:

2.3 $$\begin{aligned} \textstyle\begin{cases} dS(t)= ( \eta N -\beta I(t)S(t)-\mu S ( t ) ) \,dt-\sigma I(t) S(t)\,dB ( t ), \\ dE(t)= ( \beta I(t) S(t)-\upsilon E ( t ) -\mu E ( t ) ) \,dt+\sigma I(t) S(t)\,dB ( t ), \\ dI(t)= ( \upsilon E ( t ) -\gamma I(t)-\mu I ( t ) ) \,dt, \\ dR(t)= ( \gamma I(t)-\mu R ( t ) ) \,dt. \end{cases}\displaystyle \end{aligned}$$

The basic reproduction number $R_{0}$ is defined as the expected number of secondary cases produced by a single infection in a completely susceptible population [4, 6, 10]. In many definitions of basic reproduction number that have been proposed, the basic conceptual framework is similar. This is also called the basic reproduction ratio, which is an epidemiological metric used to describe the transmission of an infectious disease. Mathematically, the basic reproduction number is defined as follows [11].

The *basic reproduction number* of an epidemic model $R_{0}$ is given by

2.4 $$\begin{aligned} R_{0}:= \int_{0}^{+\infty } b ( a ) F ( a ) \,da\text{,} \end{aligned}$$

where $b ( a ) $ is the average number of new infected individuals (in a completely susceptible population) by an infected individual if it is infectious during all the time between 0 and *a*. $F ( a ) $ is the probability of a new infected individual continuous infecting during the time interval between 0 and *a*. This is also called the underlying *survival probability* (or *function*). Note that in the case of the SEIR model $b ( a ) =\frac{\eta }{\mu } \upsilon \beta N$ and $F ( a ) =e^{- ( \mu +\gamma ) ( \mu + \upsilon ) a}$. For SIR model, $b ( a ) =\frac{\eta }{\mu } \beta N$ and $F ( a ) =e^{- ( \mu +\gamma ) a}$. In this way, the basic reproduction numbers for SIR and SEIR models are, respectively,

2.5 $$\begin{aligned} R_{0}^{\mathrm{SIR}} = R_{0}^{\mathrm{SIS}} = \frac{\eta }{\mu } \frac{\beta N}{ ( \mu +\gamma )} \quad\text{and}\quad R_{0}^{\mathrm{SEIR}} = \frac{\eta }{\mu } \frac{\upsilon \beta N}{ ( \mu +\upsilon ) ( \mu +\gamma )}. \end{aligned}$$

See the example in Appendix A.1. The basic reproduction number is built for the SEIR model with demography. We now give some basic definitions and preliminary results for the benefit of the readers in the following subsection.

### Preliminaries and basic definitions

In this section, we introduce the basic notions and the theoretical framework that we need in this paper. The following definition of equilibrium point is given [12].

#### Definition 2.1

Let an ordinary differential system be given by

$$\begin{aligned} \dot{X} ( t ) =f \bigl( X ( t ) \bigr)\quad \text{for all } t\geq t_{0}, \end{aligned}$$

with the matrix notation

2.6 $$\begin{aligned} \begin{pmatrix} dX_{1} ( t ) / dt \\ \vdots \\ dX_{n} ( t ) / dt\end{pmatrix} = \begin{pmatrix} f_{1} ( X_{1} ( t ),\ldots,X_{n} ( t ) ) \\ \vdots \\ f_{n} ( X_{1} ( t ),\ldots,X_{n} ( t ) )\end{pmatrix}, \end{aligned}$$

where $f_{i}: \mathbb{R}^{n} \longrightarrow \mathbb{R}$ is a locally Lipschitz function for all $i=1, \ldots,n$. $\overset{\circ }{x}\in R^{n}$ is called an *equilibrium point* $f(\overset{\circ }{x})=0_{n}$, where $\mathbf{0}_{n}$ is a matrix with size $n\times 1$.

Let be an equilibrium point $\overset{\circ }{x}\in R^{n}$ of the ordinary differential system $\dot{X} ( t ) =f ( X ( t ) ) $. If $\overset{\circ }{x}$ is different to $\overset{\circ }{x}\neq X(t_{0})$, it is possible to consider the substitution $\xi (t)=X(t)-\overset{\circ }{x}$ obtaining $\dot{\xi }(t)=f(\xi (t)+\overset{\circ }{x})=f(X(t))$. In this case, the stability with respect to the point $\xi ( t_{0} ) $ [12] and the reason why the stability and the asymptotic stability are defined for the point $X ( t_{0} ) $ have been studied.

#### Definition 2.2

The point $X ( t_{0} ) $ of system (2.6) is called

(i) *Stable* if and only if, for all $\epsilon >0$, there exists $\delta >0$ such that

$$\begin{aligned} \bigl\Vert X ( t_{0} ) \bigr\Vert < \delta \quad\text{implies } \bigl\Vert X ( t ) \bigr\Vert < \epsilon \text{ for all } t>t_{0}; \end{aligned}$$

(ii) *Asymptotically stable* if and only if it is stable and can be chosen $\delta >0$ such that

$$\begin{aligned} \bigl\Vert X ( t_{0} ) \bigr\Vert < \delta \quad\text{implies } \lim_{t\rightarrow +\infty } \bigl\Vert X ( t_{0} ) \bigr\Vert =0. \end{aligned}$$

Intuitively, $X ( t_{0} ) $ is *stable* if the solutions which start *near enough* to the path which starts in $X ( t_{0} ) $ ($\Vert X ( t_{0} ) \Vert <\delta $) remain *near enough* to the path for every $t\geq t_{0}$ ($\Vert X ( t ) \Vert < \epsilon $), that is, if a solution starts near to $X ( t_{0} ) $, then it will never move away enough from the path $X ( t )$. The point is *asymptotically stable* if the solutions which start near to the path with origin in $X ( t_{0} ) $ converge to that path (see [12]).

The disease-free equilibrium point results to be locally asymptotically stable if the reproduction number is less than unity, while the endemic equilibrium point is locally asymptotically stable if such a number exceeds unity. In the deterministic epidemic models, the *disease-free equilibrium points* are locally asymptotically stable if the reproduction number is less than unity. In contrast, the endemic equilibrium point is locally asymptotically stable if the reproduction number exceeds unity (see [23]). For the SEIR model, assume $E(t)=0$ and $I(t)=0$ for any *t*, and for the models SIS and SIR, $I(t)=0$. For the deterministic case, the disease-free equilibrium points of the SIR and SEIR models with demography are $( \frac{\eta }{\mu }N,0,0 ) $ and $( \frac{\eta }{\mu }N,0,0,0 ) $, respectively. Under the SIR model we have that if $R^{\mathrm{SIR}}_{0} < 1$, then $(\frac{\eta }{\mu } N, 0, 0)$ is asymptotically stable. Note that a numerical condition on the basic reproduction number holds for the stability of the SIR model. Hence, we establish numerical conditions for which some deterministic epidemic models are asymptotically stable on the disease-free equilibrium points (for more details, see Appendix B). We now briefly discuss the stability analysis for stochastic differential equations on epidemic models with random perturbations. For more details, we recommend readers to refer to [15] and [18].

#### Definition 2.3

Let the system of stochastic differential equations be as follows:

2.7 $$\begin{aligned} \begin{aligned} &dX ( t ) =f \bigl( t,X ( t ) \bigr) \,dt+g \bigl( t,X ( t ) \bigr) \,dB ( t ),\quad t\geq 0, \\ &X ( 0 ) =x_{0},\end{aligned} \end{aligned}$$

where $f,g$ are locally Lipschitz functions from $\mathbb{R}^{n}$ to $\mathbb{R}$. We say that $\overset{\circ }{x}=X(t_{1})\in R^{n}$ for some $t_{1}\geq 0$ is an equilibrium point of the system if it holds $f(t_{1},\overset{\circ }{x})=0$.

If $\overset{\circ }{x}\neq X(0)$ is an equilibrium point, and substituting $\xi (t)=X(t)-\overset{\circ }{x}$, we have the system

$$d\xi (t)=f\left(t,\xi (t)+\overset{\circ }{x}\right)\;dt+g\left(t,\xi (t)+\overset{\circ }{x}\right)\;dB(t),$$

$\xi ( 0 ) $ is an equilibrium point. Using this, the stability and the asymptotic stability are defined as follows.

#### Definition 2.4

Let be a system defined by (2.7), for which $X ( 0 ) $ is an equilibrium point. We say that $X (0 ) $ is

(i) *Stable (in probability)* if and only if, for all $\epsilon >0$, there exists $\delta >0$ such that if $\Vert X ( 0 ) \Vert <\delta $, then

$$\begin{aligned} P \Bigl( \sup_{t\geq 0} \bigl\Vert X ( t ) \bigr\Vert \geq \epsilon \Bigr) =0; \end{aligned}$$

(ii) *Asymptotically stable* if it is stable in probability, and there exists $\delta >0$ such that if $\Vert X ( 0 ) \Vert <\delta $ then

$$\begin{aligned} P \Bigl( \lim_{t\rightarrow +\infty }X ( t ) =0 \Bigr) =1. \end{aligned}$$

#### Definition 2.5

Let $\{ X ( t ) \} _{t\geq 0}$ be an Itô process and $h ( t,x ) \in C^{2} ( [ 0,+\infty ) \times \mathbb{R} ) $. We define the *differential operator* for *h* as follows:

2.8 $$\begin{aligned} \mathcal{L} \bigl( h \bigl( X ( t ) \bigr) \bigr):= \frac{\partial h}{\partial t} \bigl( t,X ( t ) \bigr) +u(t) \frac{\partial h}{\partial x} \bigl( t,X ( t ) \bigr) + \frac{1}{2}v^{2}(t) \frac{\partial ^{2}h}{\partial x^{2}} \bigl( t,X ( t ) \bigr). \end{aligned}$$

For observing the stability in SIS and SEIR models with random perturbations, using adequate Lyapunov functions, we state now the following theorem given in [22] without proof.

#### Theorem 2.1

*Let* $V (X ( t ) ) $ *defined on* $V:\mathbb{R}^{n}\longrightarrow \mathbb{R}$ *be a Lyapunov function*.

(*i*) *If* $\mathcal{L} ( V ( X ( t ) ) ) \leq 0$ *for all* $t\geq 0$, *then* $X ( 0 ) $ *is stable in probability*.

(*ii*) *If* *V* *satisfies* (i) *and* $\mathcal{L} ( V ( X ( t ) ) ) <0$, *then* $X (t ) $ *is asymptotically stable*.

We prove the following theorem by constructing a Lyapunov function and give the sufficient conditions at which the point $( \frac{\eta }{\mu }N,0,0,0 ) $ is asymptotically stable in the SEIR model with random perturbations. In [17] the author used a similar approach for constructing a Lyapunov function to prove that the endemic equilibrium state is globally asymptotically stable.

#### Theorem 2.2

*If the parameters of the SEIR model with random perturbations satisfy the following*:

2.9 $$\begin{aligned} 0< \upsilon \beta \frac{\eta N}{\mu }< ( \gamma +\mu ) ( \upsilon + \mu ) - \frac{\sigma ^{2}\upsilon ^{2}\eta ^{2}N^{2}}{2\mu ^{2}} \end{aligned}$$

*and* $\upsilon +\mu >1$, *then the point* $( \frac{\eta }{\mu }N,0,0,0 ) $ *is asymptotically stable*.

#### Proof

Let the function be given by

$$\begin{aligned} W ( S,E,I,R ):=\lambda _{1} \biggl(\frac{\eta }{\mu } N-S \biggr) ^{2}+\lambda _{2} \biggl( \upsilon EI+\upsilon ^{2} \frac{1 }{2}E^{2}+ ( \mu +\upsilon ) \frac{1 }{2 }I^{2} \biggr) + \frac{1 }{2}\lambda _{3}R^{2}, \end{aligned}$$

where $\lambda _{1},\lambda _{2},\lambda _{3}>0$ are adequately chosen. As $V (S,E,I,R ) >0$ for all $t>0$ and $V (\frac{\eta }{\mu }N,0,0,0 ) =0$. In addition, the partial derivatives of *V* are continuous, therefore *V* is a Lyapunov function.

We rewrite in the matrix form $dx ( t ) =f ( t,x ( t ) ) \,dt+g ( t,x ( t ) ) \,dB ( t ) $, with $x ( t ):= ( S ( t ),E ( t ), I ( t ),R ( t ) ) $, and *f*, *g* given by

$$\begin{aligned} &f^{T}= \bigl[ f \bigl( t,x ( t ) \bigr) \bigr] ^{T}:= \bigl( \eta N -\beta SI-\mu S, \beta SI- ( \upsilon +\mu ) E, \upsilon E- ( \mu +\gamma ) I, \gamma I-\mu R \bigr) \quad\text{and}\\ & g^{T}= \bigl[ g \bigl( t,x ( t ) \bigr) \bigr] ^{T}:= \begin{pmatrix} -\sigma S ( t ) I ( t ), & \sigma S ( t ) I ( t ), & 0, & 0\end{pmatrix}. \end{aligned}$$

For calculating $\mathcal{L} ( V ( t ) ) $, we have

$$\begin{aligned} f^{T} \frac{\partial W}{\partial x} = {}& \begin{pmatrix} \eta N -\beta SI-\mu S, & \beta SI- ( \upsilon +\mu ) E, & \upsilon E- ( \mu +\gamma ) I, & \gamma I-\mu R\end{pmatrix} A \\ = {}&{ -}2\lambda _{1} \biggl( \frac{\eta }{\mu }N-S \biggr) ( \eta N - \beta IS-\mu S ) +\lambda _{2} \bigl( \bigl[ \upsilon ^{2} \beta S-\upsilon ( \gamma +\mu ) \bigr] EI \\ & {} + \bigl( \upsilon ^{2}-\upsilon ^{2} ( \upsilon + \mu ) \bigr) E^{2}+ \bigl[ \upsilon \beta S- ( \gamma + \mu ) ( \mu + \upsilon ) \bigr] I^{2} \bigr) + \lambda _{3} \bigl( \gamma RI-\mu R^{2} \bigr), \end{aligned}$$

where

$$\begin{aligned} A= \begin{pmatrix} -2\lambda _{1} ( \frac{\eta }{\mu } N-S ), & \lambda _{2} ( \upsilon ^{2}E+\upsilon I ), & \lambda _{2} ( \upsilon E+ ( \mu +\upsilon ) I ), & \lambda _{3}R\end{pmatrix} ^{T}. \end{aligned}$$

On the other hand, when $\eta \geq \mu $ we have

$$\begin{aligned} \frac{1}{2}g^{T} \frac{\partial V}{\partial x}g & = \frac{1 }{2}\sigma ^{2}S^{2}I^{2} \begin{pmatrix} -1, & 1, & 0, & 0\end{pmatrix} \begin{pmatrix} 2\lambda _{1} & 0 & 0 & 0 \\ 0 & \lambda _{2}\upsilon ^{2} & \lambda _{2}\upsilon & 0 \\ 0 & \lambda _{2}\upsilon & \lambda _{2} ( \mu +\upsilon ) & 0 \\ 0 & 0 & 0 & 2\lambda _{4} \end{pmatrix} \begin{pmatrix} -1 \\ 1 \\ 0 \\ 0 \end{pmatrix} \\ & = \frac{1 }{2}\sigma ^{2}S^{2}I^{2} \begin{pmatrix} -2\lambda _{1}, & \lambda _{2}\upsilon ^{2}, & \lambda _{2}\upsilon, & 0\end{pmatrix} \begin{pmatrix} -1, & 1, & 0, & 0\end{pmatrix} ^{T} \\ & =\lambda _{1}\sigma ^{2}S^{2}I^{2}+ \frac{1 }{2}\lambda _{2}\upsilon ^{2}\sigma ^{2}S^{2}I^{2}\leq \lambda _{1} \sigma ^{2}S^{2}I^{2}+\lambda _{2} \frac{1 }{2}\upsilon ^{2}\sigma ^{2} \frac{\eta ^{2} N^{2}}{ \mu ^{2} }I^{2} , \end{aligned}$$

therefore

$$\begin{aligned} \mathcal{L} \bigl( W ( t ) \bigr) =f^{T} \frac{\partial V}{\partial x}+ \frac{406}{2}g^{T} \frac{\partial V}{\partial x}g\leq \lambda _{1}a(t)+\lambda _{2}b(t)+\lambda _{3}c(t), \end{aligned}$$

such that

$$\begin{aligned} &a(t)= 2 \biggl(\frac{\eta }{\mu }N-S \biggr) ( -\eta N +\beta IS+\mu S ) +\sigma ^{2}S^{2}I^{2}, \\ &b ( t ) = \biggl( \biggl( \upsilon ^{2}\beta \frac{\eta }{\mu }N- \upsilon ( \upsilon +\mu ) \biggr) + \bigl( \upsilon ^{2}-\upsilon ^{2} ( \upsilon +\mu ) \bigr) \\ &\phantom{b ( t ) =}{}+ \biggl( \upsilon \beta \frac{\eta }{\mu }N- ( \mu +\gamma ) ( \mu + \upsilon ) +\frac{1 }{ 2 }\upsilon ^{2}\sigma ^{2} \frac{\eta ^{2}}{\mu ^{2}} N^{2} \biggr) \biggr) \inf _{t\geq 0} \bigl\{ EI, E^{2}, I^{2} \bigr\} , \\ &c ( t ) = \gamma IR-\mu R^{2}. \end{aligned}$$

See (i) of the proof for Theorem B.2, it is clear that $\upsilon ^{2}\beta \frac{\eta }{\mu }N-\upsilon ( \upsilon + \mu ) + \upsilon ^{2}-\upsilon ^{2} ( \upsilon +\mu ) < 0$.

On the other hand, as $\upsilon \beta \frac{\eta }{\mu }N + \frac{ 1 }{ 2 }\upsilon ^{2}\sigma ^{2} \frac{\eta ^{2}}{\mu ^{2}} N^{2} < ( \mu +\gamma ) ( \mu +\upsilon )$, then

$$\begin{aligned} \upsilon \beta \frac{\eta }{\mu }N- ( \mu +\gamma ) ( \mu +\upsilon ) + \frac{1 }{ 2 }\upsilon ^{2}\sigma ^{2} \frac{\eta ^{2}}{\mu ^{2}} N^{2} < 0, \end{aligned}$$

therefore $b(t) < 0$. If $\eta < \mu $, the proof is analogous to Theorem B.2, having $t_{0}> 0$ for which $b(t)<0$ for any $t>t_{0}$.

Choosing adequately $\lambda _{1}$, $\lambda _{2}$, and $\lambda _{3}$, for any case, it has that

$$\begin{aligned} \mathcal{L} \bigl( W ( t ) \bigr) = \leq \lambda _{1}a(t)+ \lambda _{2}b(t)+\lambda _{3}c(t) < 0 \end{aligned}$$

for all $t>t_{0}$, showing that the point $( \frac{\eta }{\mu }N,0,0,0 ) $ is asymptotically stable. □

#### Theorem 2.3

*If the parameters of the SIS model with random perturbation satisfy that*

2.10 $$\begin{aligned} 0< \beta \frac{\eta N}{\mu }< \gamma +\mu - \frac{\sigma ^{2}\eta ^{2}N^{2}}{2\mu ^{2}}, \end{aligned}$$

*then the point* $( \frac{\eta }{\mu } N,0 ) $ *is asymptotically stable*.

#### Proof

The proof is similar to the previous theorem. Take *V* defined by

$$\begin{aligned} V \bigl( S ( t ),I ( t ) \bigr):=\lambda _{1} \biggl( \frac{\eta }{\mu }N-S ( t ) \biggr) ^{2}+ \frac{1}{2}\lambda _{2}I^{2} ( t ), \end{aligned}$$

where $\lambda _{1},\lambda _{1}>0$ are positive constants adequately chosen. □

Theoretically, by inequality (2.10) it is shown that (Theorem 2.3) if

2.11 $$\begin{aligned} \beta \frac{\eta N}{\mu (\gamma +\mu ) } + \frac{\sigma ^{2}\eta ^{2}N^{2}}{2\mu ^{2} (\gamma +\mu ) } < 1, \end{aligned}$$

then the point $( \frac{\eta }{\mu } N,0 ) $ is asymptotically stable.

According to Theorem 2.2, that $( \frac{\eta }{\mu } N, 0, 0, 0 ) $ in the SEIR model with random perturbations is asymptotically stable, and it is necessary that $\mu + \upsilon >1$ and inequality (2.9) hold and can be written as

2.12 $$\begin{aligned} \upsilon \beta \frac{\eta N}{\mu (\gamma +\mu ) (\upsilon +\mu ) } + \frac{\sigma ^{2} \eta ^{2} \upsilon ^{2} N^{2}}{2\mu ^{2} (\gamma +\mu ) (\upsilon +\mu ) } < 1. \end{aligned}$$

## Simulation results for the stability of the stochastic models

In this section, we discuss simulation results of the reproduction numbers $R^{\mathrm{SIR}}_{0,E}, R^{\mathrm{SIS}}_{0,E}$, and $R^{\mathrm{SEIR}}_{0,E}$ respectively for SIR, SIS, and SEIR models with random perturbations. Our objective is to find the *smallest* value of $R^{\mathrm{SIS}}_{0,E}$ such that $R^{\mathrm{SIS}}_{0,E}<1$ and for which the SIS model with random perturbation is asymptotically stable on $( \frac{\eta }{\mu } N,0 )$ (according to Theorem 2.3). Similarly, we search for the *smallest* value of $R^{\mathrm{SEIR}}_{0,E}$ such that $R^{\mathrm{SEIR}}_{0,E}<1$ and $( \frac{\eta }{\mu } N,0 ) $ is asymptotically stable on the SEIR model with random perturbations (according to Theorem 2.2). We now observe through simulations the smallest values of $R^{\mathrm{SIS}}_{0,E}$ and $R^{\mathrm{SEIR}}_{0,E}$ for which the asymptotic stability holds.

We now apply the *Euler–Maruyama method* for simulating the SIS and SEIR models with random perturbations [21]. The approximation equations of the models are given by

3.1 $$\begin{aligned} &\textstyle\begin{cases} S ( t_{j+1} ) = S ( t_{j} ) + [ \eta N-\beta S ( t_{j} ) I ( t_{j} ) -\mu S ( t_{j} ) + \gamma I ( t_{j} ) ] ( t_{j+1}-t_{j} ) \\ \phantom{S ( t_{j+1} ) =}{}-\sigma S ( t_{j} ) I ( t_{j} ) ( B ( t_{j+1} ) -B ( t_{j} ) ), \\ I ( t_{j+1} ) =I ( t_{j} ) + [ \upsilon E ( t_{j} ) - ( \mu +\gamma ) I ( t_{j} ) ] ( t_{j+1}-t_{j} ), \end{cases}\displaystyle \end{aligned}$$

3.2 $$\begin{aligned} &\textstyle\begin{cases} S ( t_{j+1} ) = S ( t_{j} ) + [ \eta N-\beta S ( t_{j} ) I ( t_{j} ) -\mu S ( t_{j} ) ] ( t_{j+1}-t_{j} )\\ \phantom{S ( t_{j+1} ) =}{} -\sigma S ( t_{j} ) I ( t_{j} ) ( B ( t_{j+1} ) -B ( t_{j} ) ),\\ E ( t_{j+1} ) = E ( t_{j} ) + [ \beta S ( t_{j} ) I ( t_{j} ) - ( \upsilon +\mu ) E ( t_{j} ) ] ( t_{j+1}-t_{j} ) \\ \phantom{E ( t_{j+1} ) =}{}+\sigma S ( t_{j} ) I ( t_{j} ) ( B ( t_{j+1} ) -B ( t_{j} ) ),\\ I ( t_{j+1} ) =I ( t_{j} ) + [ \upsilon E ( t_{j} ) - ( \mu +\gamma ) I ( t_{j} ) ] ( t_{j+1}-t_{j} ),\\ R ( t_{j+1} ) =R ( t_{j} ) + [ \gamma I ( t_{j} ) -\mu R ( t_{j} ) ] ( t_{j+1}-t_{j} ).\end{cases}\displaystyle \end{aligned}$$

**Figure 1:**
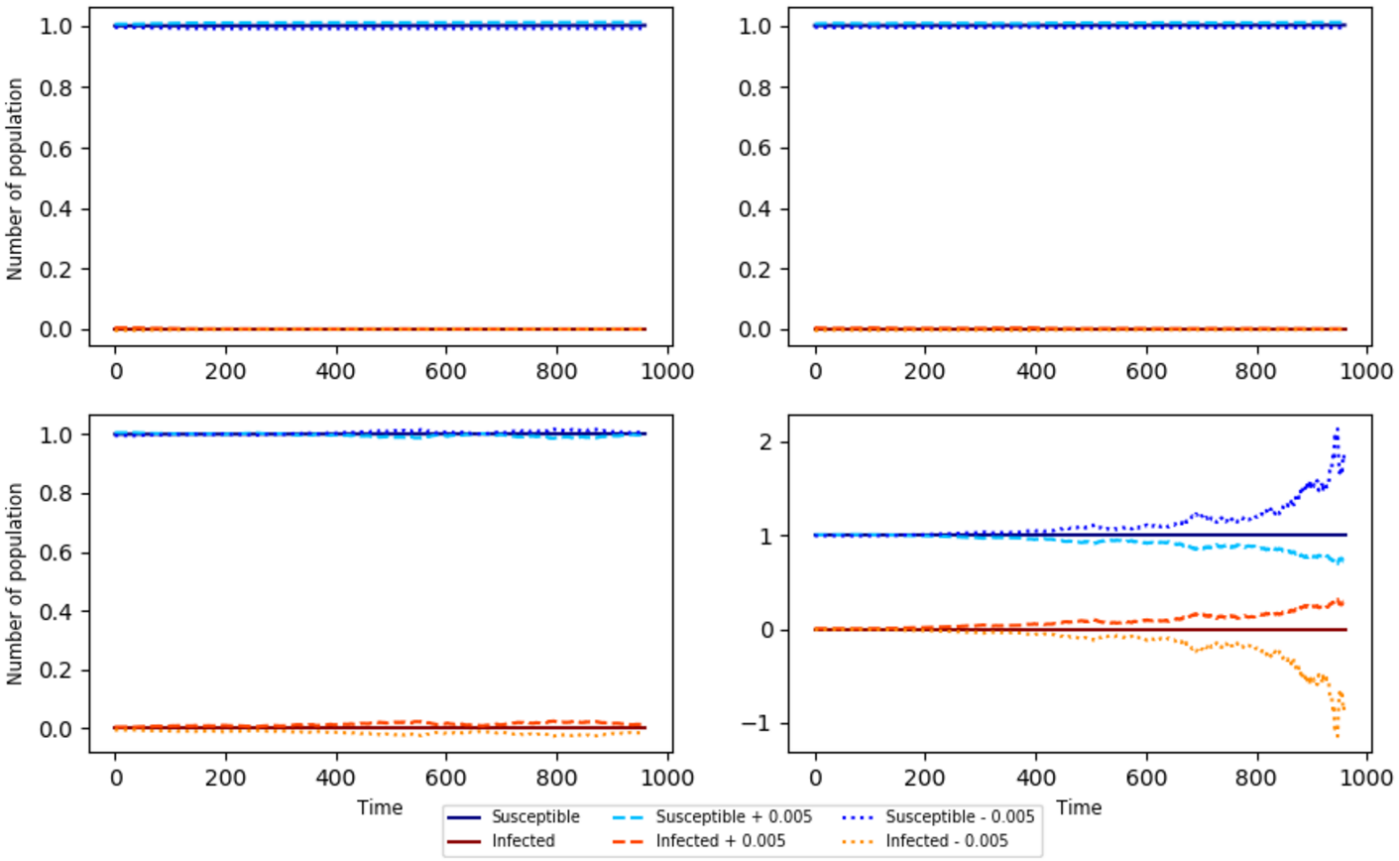
Stability modeled using the parameters $N=1$, $\beta = 0.5$, $\sigma = 0.3$, $\mu = 0.0007$, $\eta = 0.0008$, and **(a)** $\gamma = 0.6$ (upper right), **(b)** $\gamma = 0.7$ (upper left), **(c)** $\gamma = \beta N \frac{\eta }{\mu } - \frac{\sigma ^{2}}{2} \frac{\eta ^{2}}{\mu ^{2}} N^{2} - \mu $ (lower left) and (**d**) $\gamma = 0.2$ (lower right). The initial condition is $( N,0 ) = ( 1,0 )$ for all of them

The numeric conditions for which the *disease-free equilibrium* $( \frac{\eta }{\mu }N,0 ) $ for the simulations presented at the point $( \frac{0.0008 }{0.0007 }1,0 )$) on the SIS model with random perturbation is asymptotically stable. Note that when $R^{\mathrm{SIS}}_{0,e}=\beta N \frac{\eta }{\mu (\gamma + \mu )} + \frac{\sigma ^{2}}{2} \frac{\eta ^{2} N^{2}}{\mu ^{2} (\gamma + \mu )} < 1$ (see Fig. 1, upper left) the asymptotic stability is clear since the functions remain “near” to the constant functions $y=\frac{\eta }{\mu }N$ and $y=0$, varying these functions +0.0005 and −0.0005. Similarly, the asymptotic stability is observed when $R^{\mathrm{SIS}}_{0,e} >1$ and $\beta N \frac{\eta }{\mu (\gamma + \mu )} - \frac{\sigma ^{2}}{2} \frac{\eta ^{2} N^{2}}{\mu ^{2} (\gamma + \mu )} < 1$ (Fig. 1, upper right). When $\beta N \frac{\eta }{\mu (\gamma + \mu )} - \frac{\sigma ^{2}}{2} \frac{\eta ^{2} N^{2}}{\mu ^{2} (\gamma + \mu )} = 1$ (Fig. 1, lower left), the stability is not so clear, while it is clear when $\beta N \frac{\eta }{\mu (\gamma + \mu )} - \frac{\sigma ^{2}}{2} \frac{\eta ^{2} N^{2}}{\mu ^{2} (\gamma + \mu )} > 1$ (Fig. 1, lower right). We observe that as $\beta N \frac{\eta }{\mu (\gamma + \mu )} - \frac{\sigma ^{2}}{2} \frac{\eta ^{2} N^{2}}{\mu ^{2} (\gamma + \mu )} < 1$ guarantees the asymptotic stability for the disease-free equilibrium, based on the simulation results, we propose the following conjecture.

### Conjecture 3.1

*If*

3.3 $$\begin{aligned} R^{\mathrm{SIS}}_{0,E}:=\beta \frac{\eta N}{\mu (\gamma +\mu ) } - \frac{\sigma ^{2}\eta ^{2}N^{2}}{2\mu ^{2} (\gamma +\mu ) } < 1, \end{aligned}$$

*then* $( \frac{\eta }{\mu }N,0 )$ *is asymptotically stable on the SIS model with random perturbation*.

Now, we focus our attention on the simulations of the stability for the SEIR model with random perturbations which are shown for determining the numeric conditions under which the point $( \frac{\eta }{\mu }N,0, 0, 0 ) $ is asymptotically stable on the SEIR model with random perturbations, for example, the values of $( \frac{0.9 }{0.4 }1, 0, 0, 0 ) $) are verified numerically.

**Figure 2:**
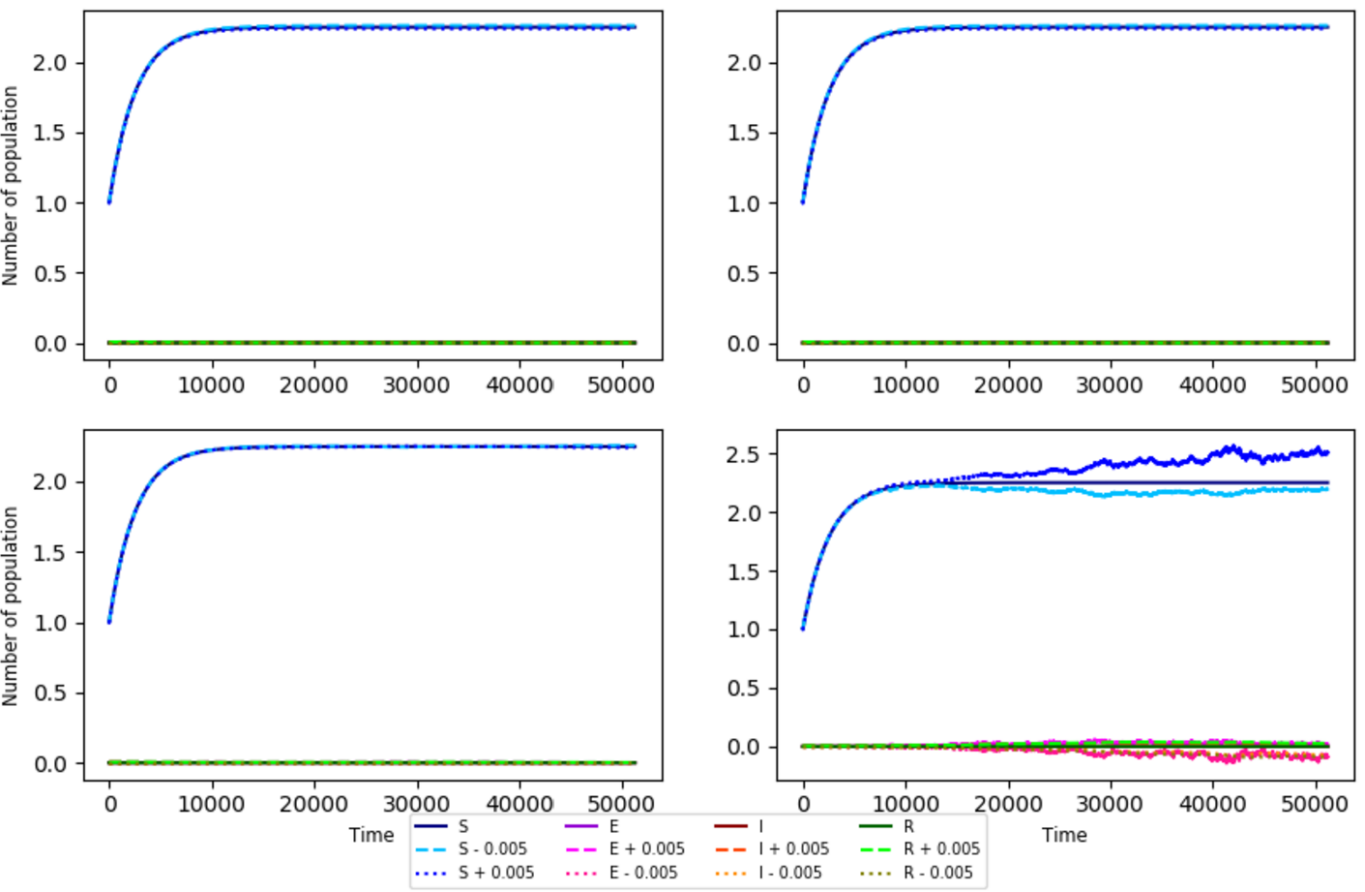
Stability modeled using the parameters $N=1$, $\beta = 0.8$, $\sigma = 0.3$, $\mu = 0.4$, $\eta = 0.9$, and **(a)** $\gamma = 0.85$ and $\upsilon = 0.7$ (upper right), **(b)** $\gamma = 0.75$ and $\upsilon = 0.7$ (upper left), **(c)** $\gamma = \beta \upsilon N \frac{\eta }{\mu (\mu + \upsilon )} - \frac{\sigma ^{2}}{2} \frac{\upsilon ^{2} \eta ^{2}}{\mu ^{2} (\mu + \upsilon )} N^{2} - \mu $ and $\upsilon = 0.85$ (lower left) and (**d**) $\gamma = 0.55$ and $\upsilon = 0.85$ (lower right). The initial condition is $( N,0, 0, 0 ) = ( 1,0, 0, 0 )$ for all of them

In all of the previous simulations, we assume that $\upsilon + \mu >1$. Note that when $R^{\mathrm{SEIR}}_{0,e}= \frac{\upsilon \beta \eta N}{\mu (\gamma + \mu )(\upsilon + \mu )} + \frac{\upsilon ^{2} \sigma ^{2}}{2} \frac{\eta ^{2} N^{2}}{\mu ^{2} (\gamma + \mu )(\upsilon + \mu )} < 1$ (see Fig. 2, upper left) the asymptotic stability is clear since the functions remain “near” to the constant functions $y=\frac{\eta }{\mu }N$ and $y=0$, varying these functions +0.0005 and −0.0005. Similarly, the asymptotic stability is observed when $R^{\mathrm{SEIR}}_{0,e} >1$ and $\frac{\upsilon \beta \eta N}{\mu (\gamma + \mu )(\upsilon + \mu )} - \frac{\upsilon ^{2} \sigma ^{2}}{2} \frac{\eta ^{2} N^{2}}{\mu ^{2} (\gamma + \mu )(\upsilon + \mu )} < 1$ (Fig. 2, upper right). When $\frac{\upsilon \beta \eta N}{\mu (\gamma + \mu )(\upsilon + \mu )} - \frac{\upsilon ^{2} \sigma ^{2}}{2} \frac{\eta ^{2} N^{2}}{\mu ^{2} (\gamma + \mu )(\upsilon + \mu )} = 1$ (Fig. 2, lower left), the instability is not so clear, while the instability is clear when $\frac{\upsilon \beta \eta N}{\mu (\gamma + \mu )(\upsilon + \mu )} - \frac{\upsilon ^{2} \sigma ^{2}}{2} \frac{\eta ^{2} N^{2}}{\mu ^{2} (\gamma + \mu )(\upsilon + \mu )} > 1$ (Fig. 2, lower right) since it is observed that the varied solutions move away from the disease-free equilibrium. As $\frac{\upsilon \beta \eta N}{\mu (\gamma + \mu )(\upsilon + \mu )} - \frac{\upsilon ^{2} \sigma ^{2}}{2} \frac{\eta ^{2} N^{2}}{\mu ^{2} (\gamma + \mu )(\upsilon + \mu )} < 1$ and $\upsilon + \mu >1$ guarantee the asymptotic stability for the disease-free equilibrium (according to the simulations), we now propose the conjecture.

### Conjecture 3.2

*If* $\upsilon + \mu >1$ *and*

3.4 $$\begin{aligned} R^{\mathrm{SEIR}}_{0,E}:= \frac{\upsilon \beta \eta N}{\mu (\gamma +\mu ) (\upsilon +\mu ) } - \frac{\sigma ^{2} \eta ^{2} \upsilon ^{2} N^{2}}{2\mu ^{2} (\gamma +\mu ) (\upsilon +\mu ) } < 1, \end{aligned}$$

*then* $( \frac{\eta }{\mu }N,0, 0, 0 )$ *is asymptotically stable on the SEIR model with random perturbations*.

**Figure 3:**
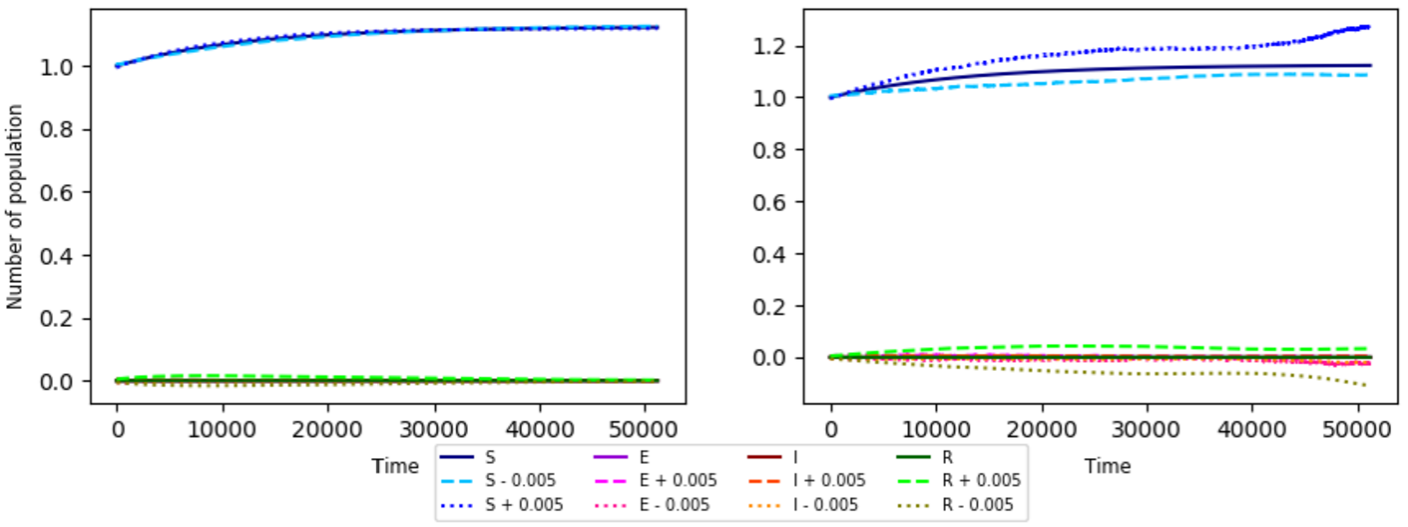
Stability modeled using the parameters $N=1$, $\beta = 0.8$, $\sigma = 0.3$, $\mu = 0.08$, $\eta = 0.09$, and **(a)** $\gamma = 0.85$ and $\upsilon = 0.7$ (right), **(b)** $\gamma = 0.75$ and $\upsilon = 0.7$ (left). The initial condition is $( N,0, 0, 0 ) = ( 1,0, 0, 0 )$ for all of them

As the basic reproduction number of the SEIR model with random perturbations $R^{\mathrm{SEIR}}_{0,E}$ (with $R^{\mathrm{SEIR}}_{0,E}<1$, $\upsilon + \mu >1$) is the lower number for which $( \frac{\eta }{\mu }N,0, 0, 0 )$ is asymptotically stable. In the Fig. 3, we show that the condition $\upsilon + \mu >1$ is not satisfied.

It is clear that despite of being $R^{\mathrm{SEIR}}_{0,e} < 1$, if $\mu + \upsilon <1$, the stability is not so clear. Similarly, if $R^{\mathrm{SEIR}}_{0,E} < 1$ and $\mu + \upsilon <1$, according to the simulation, the point $( \frac{\eta }{\mu }N,0, 0, 0 )$ (in this case $\frac{\eta }{\mu }N=\frac{0.09 }{0.08}N$) is unstable. But it is important to have the condition $\mu + \upsilon >1$ for retaining the asymptotic stability on the SEIR model with random perturbations.

We wish to note that, for the SIR model with random perturbation, the following inequality holds for having the asymptotic stability in $( \frac{\eta }{\mu } N, 0, 0 ) $ for the model proposed in [25] and [28]

3.5 $$\begin{aligned} R^{\mathrm{SIR}}_{0,E}:=\beta \frac{\eta N}{\mu (\gamma +\mu ) } - \frac{\sigma ^{2}\eta ^{2}N^{2}}{2\mu ^{2} (\gamma +\mu ) } < 1. \end{aligned}$$

### Basic reproduction variable and their statistical tests

We now study the basic reproduction number as a normally distributed random variable. For the deterministic model, $R_{0}$ is defined in integral (2.4). Consider the SIR model with random perturbation, the *survival integral* is given by

3.6 $$\begin{aligned} R_{0,v}^{\mathrm{SIR}}:= \int_{0}^{{+\infty } } \bigl( \beta + \sigma B ( a ) \bigr) Ne^{- ( \mu +\gamma ) a}\,da, \end{aligned}$$

where $R_{0,v}^{\mathrm{SIR}}$ is a normally distributed random variable. We refer the reader to consult (A.1) for the SEIR deterministic model. Set $F(a)=e^{-(\mu +\gamma )a}$, from the above equation, $R_{0,v}^{\mathrm{SIR}}$ is given by

$$\begin{aligned} R_{0,v}^{\mathrm{SIR}}&= \int_{0}^{{+\infty }} \bigl( \beta +\sigma B ( a ) \bigr) Ne^{- ( \mu +\gamma ) a}\,da=\int_{0}^{{+\infty } }\beta Ne^{- ( \mu +\gamma ) a}\,da+\sigma \int_{0}^{{+\infty } }B ( a ) Ne^{- ( \mu +\gamma ) a}\,da \\ &=\frac{\beta N}{ ( \mu +\gamma )}+\sigma N \int_{0}^{{+\infty } }B ( a ) e^{- ( \mu +\gamma ) a} \,da, \end{aligned}$$

using the integration-by-parts rule [19], we have an expression which involves $\int ^{+ \infty }_{0}B(a) e^{- (\mu +\gamma ) a}\,da$ given by

$$\begin{aligned} \lim_{l\rightarrow +\infty } B ( l ) e^{- ( \mu +\gamma ) l}=B ( 0 ) - ( \mu +\gamma ) \int_{0}^{{+\infty } }B ( a ) e^{- ( \mu +\gamma ) a}\,da+ \int_{0}{^{+\infty } }e^{- ( \mu + \gamma ) a}\,dB ( a ), \end{aligned}$$

where $\{ B ( t ) \} _{t\geq 0}$ is a Brownian motion, thus

$$\begin{aligned} \int_{0}^{{+\infty } } \frac{e^{- ( \mu +\gamma ) a}}{\mu +\gamma }\,dB ( a ) - \frac{1 }{\mu +\gamma }\lim_{l\rightarrow +\infty } B ( l ) e^{- ( \mu +\gamma ) l}= \int_{0}^{{+\infty } }B ( a ) e^{- ( \mu +\gamma ) a}\,da. \end{aligned}$$

The above integral $\int _{0}^{l}B ( a ) e^{- ( \mu +\gamma ) a}\,da$ is well defined, we get (see [16, p. 393])

3.7 $$\begin{aligned} \int_{0}^{{+\infty } }B ( a ) e^{- ( \mu +\gamma ) a}\,da \sim \mathbf{N} \biggl( - \frac{1 }{\mu +\gamma }\lim_{l\rightarrow +\infty }B ( l ) e^{- ( \mu +\gamma ) l}, \int_{0}^{{+\infty } } \frac{e^{-2 ( \mu +\gamma ) a}}{ ( \mu +\gamma ) ^{2}}\,da \biggr). \end{aligned}$$

By the *law of the iterated logarithm* [1, p. 66], we get

$$\begin{aligned} \mathop{\lim \sup }_{l\rightarrow +\infty } \frac{B ( l )}{\sqrt{2l\log \log l}}=1\quad \text{a.s.}, \end{aligned}$$

we have

3.8 $$\begin{aligned} 0&\leq \lim_{l\rightarrow +\infty } B ( l ) e^{- ( \mu +\gamma ) l}\leq \mathop{\lim \sup }_{l\rightarrow +\infty } \frac{B ( l ) \sqrt{2l\log \log t}e^{- ( \mu +\gamma ) l}}{\sqrt{2l\log \log l}} \\ &=\mathop{\lim \sup }_{l\rightarrow +\infty }\sqrt{2l \log \log t}e^{- ( \mu +\gamma ) l} \quad\text{a.s.} \end{aligned}$$

On the other hand, we have

$$\begin{aligned} 0\leq \lim_{l\rightarrow +\infty } \sqrt{2l\log \log t}e^{- ( \mu +\gamma ) l}\leq \lim _{l\rightarrow +\infty } \sqrt{2l\log l}e^{- ( \mu +\gamma ) l}\leq \lim _{l\rightarrow +\infty } \sqrt{2}le^{- ( \mu + \gamma ) l}, \end{aligned}$$

and by applying the *L’Hôpital’s rule*

$$\begin{aligned} \lim_{l\rightarrow +\infty } \sqrt{2}le^{- ( \mu + \gamma ) l}=\sqrt{2}\lim _{l\rightarrow +\infty } \frac{l}{\frac{1}{e^{- ( \mu +\gamma ) l}}}=\sqrt{2}\lim_{l\rightarrow +\infty } \frac{1 }{ ( \mu +\gamma ) \frac{e^{- ( \mu +\gamma ) l}}{e^{-2 ( \mu +\gamma ) l}}}=0, \end{aligned}$$

thus,

$$\begin{aligned} \lim_{l\rightarrow +\infty } \sqrt{2l\log \log t}e^{- ( \mu +\gamma ) l}=0, \end{aligned}$$

then inequality (3.8) can be written as

$$\begin{aligned} 0&\leq \lim_{l\rightarrow +\infty } B ( l ) e^{- ( \mu +\gamma ) l}\leq \mathop{\lim \sup }_{l\rightarrow +\infty }\sqrt{2l\log \log t}e^{- ( \mu +\gamma ) l}\\ &= \lim _{l\rightarrow +\infty } \sqrt{2l\log \log t}e^{- ( \mu +\gamma ) l}=0 \quad\text{a.s.}, \end{aligned}$$

which means that $-\frac{1}{\mu +\gamma }\lim_{l\rightarrow +\infty }B ( l ) e^{- ( \mu +\gamma ) l}=0$ a.s. We see that

$$\begin{aligned} \int_{0}^{{+\infty } } \frac{e^{-2 ( \mu +\gamma ) a}}{ ( \mu +\gamma ) ^{2}}\,da=\lim _{l\rightarrow +\infty } - \frac{e^{-2 ( \mu +\gamma ) a}}{2 ( \mu +\gamma ) ^{3}} \bigg\vert _{0}^{l}= \frac{1 }{2 ( \mu +\gamma ) ^{3}}. \end{aligned}$$

Then $R_{0,v}^{\mathrm{SIR}}$ is the *random basic reproduction variable* on the SIR model with random perturbation and is given by

3.9 $$\begin{aligned} R_{0,v}^{\mathrm{SIR}}\sim \mathbf{N} \biggl( \frac{\beta N}{ ( \mu +\gamma )}, \frac{\sigma ^{2}N^{2}}{2 ( \mu +\gamma ) ^{3}} \biggr). \end{aligned}$$

Similarly, we assume that *random basic reproduction variables* on the SIS and SEIR models with random perturbations are normally distributed and are given as follows.

#### Definition 1

3.10 $$\begin{aligned} &R_{0,v}^{\mathrm{SIS}}\sim \mathbf{N} \biggl( R_{0}^{\mathrm{SIS}}, \frac{\sigma ^{2}\eta ^{2}N^{2}}{2\mu ^{2} ( \mu +\gamma ) ^{3}} \biggr) , \end{aligned}$$

3.11 $$\begin{aligned} &R_{0,v}^{\mathrm{SEIR}}\sim \mathbf{N} \biggl( R_{0}^{\mathrm{SEIR}}, \frac{\eta ^{2}\upsilon ^{2}\sigma ^{2}N^{2}}{2\mu ^{2} ( ( \mu +\upsilon ) ( \mu +\gamma ) ) ^{3}} \biggr). \end{aligned}$$

From definition (1) and inequalities (2.10) and (2.9), the following inequalities hold:

3.12 $$\begin{aligned} &R_{0,E}^{\mathrm{SIS}}\leq \mathbb{E} \bigl[ R_{0,v}^{\mathrm{SIS}} \bigr] \leq \frac{\beta \eta N}{\mu (\gamma +\mu ) } + \frac{\sigma ^{2}\eta ^{2}N^{2}}{2\mu ^{2} (\gamma +\mu ) }, \end{aligned}$$

3.13 $$\begin{aligned} &R_{0,E}^{\mathrm{SEIR}}\leq \mathbb{E} \bigl[ R_{0,v}^{\mathrm{SEIR}} \bigr] \leq \frac{\upsilon \beta \eta N}{\mu (\gamma +\mu ) (\upsilon +\mu ) } + \frac{\sigma ^{2} \eta ^{2} \upsilon ^{2} N^{2}}{2\mu ^{2} (\gamma +\mu ) (\upsilon +\mu ) }. \end{aligned}$$

Note that

$$\begin{aligned} p&= P \biggl( R_{0,E}^{\mathrm{SEIR}}\leq R_{0,v}^{\mathrm{SEIR}} \leq \upsilon \beta \frac{\eta N}{\mu (\gamma +\mu ) (\upsilon +\mu )} + \frac{\sigma ^{2} \eta ^{2} \upsilon ^{2} N^{2}}{2\mu ^{2} (\gamma +\mu ) (\upsilon +\mu )} \biggr) \\ &= P \biggl( \frac{R_{0,E}^{\mathrm{SEIR}}-\mathbb{E} [ R_{0,v}^{\mathrm{SEIR}} ] }{ \sqrt{\mathbb{V} [ R_{0,v}^{\mathrm{SEIR}} ] }}\leq \frac{R_{0,v}^{\mathrm{SEIR}}-\mathbb{E} [ R_{0,v}^{\mathrm{SEIR}} ]}{\sqrt{\mathbb{V} [ R_{0,v}^{\mathrm{SEIR}} ] }}\leq \frac{\frac{\upsilon \beta \eta N}{\mu (\gamma +\mu ) (\upsilon +\mu )} + \frac{\sigma ^{2} \eta ^{2} \upsilon ^{2} N^{2}}{2\mu ^{2} (\gamma +\mu ) (\upsilon +\mu )} -\mathbb{E} [ R_{0,v}^{\mathrm{SEIR}} ]}{\sqrt{\mathbb{V} [ R_{0,v}^{\mathrm{SEIR}} ] }} \biggr) \\ &= P \biggl( -\frac{\sigma \eta N\sqrt{(\mu +\gamma )(\mu +\upsilon )}}{\sqrt{2}\mu }\leq Z\leq \frac{\sigma \eta N\sqrt{(\mu +\gamma )(\mu +\upsilon )}}{\sqrt{2}\mu } \biggr) \\ &= 2\Phi \biggl( \frac{\sigma \eta N\sqrt{(\mu +\gamma )(\mu +\upsilon )}}{\sqrt{2}\mu } \biggr) -1, \end{aligned}$$

where $\Phi ( \cdot ) $ is the *distribution function* of *Z* such that $Z \sim N(0,1)$. The probability *p* satisfies

$$\begin{aligned} 0\leq 2\Phi \biggl( \frac{\sigma \eta N\sqrt{(\mu +\gamma )(\mu +\upsilon ) }}{\sqrt{2}\mu } \biggr) -1\leq 1, \end{aligned}$$

that is,

$$\begin{aligned} \frac{1 }{2}\leq \Phi \biggl( \frac{\sigma \eta N\sqrt{(\mu +\gamma )(\mu +\upsilon )}}{\sqrt{2}\mu } \biggr) \leq 1, \end{aligned}$$

this inequality holds if and only if $\sigma \eta N\frac{1}{\mu }\sqrt{ \frac{(\mu +\gamma )(\mu +\upsilon ) }{2}} \geq 0$.

On the other hand, if *μ* tends to 0, then^1^$\sigma \eta N\frac{1}{\mu }\sqrt{ \frac{(\mu +\gamma )(\mu +\upsilon ) }{2}} \rightarrow +\infty $, therefore,

$$\begin{aligned} \Phi \biggl( \sigma \eta N\frac{1}{\mu }\sqrt{ \frac{(\mu +\gamma )(\mu +\upsilon )}{2}} \biggr) \rightarrow 1, \end{aligned}$$

which means $p \rightarrow 1$. This means that when the emigration rate is lower, the random variable $R_{0,v}^{\mathrm{SEIR}}$ is closer to the number $R_{0,E}^{\mathrm{SEIR}}$. If $\mu \rightarrow +\infty $, then^2^

$$\begin{aligned} \Phi \biggl( \sigma \eta N\frac{1}{\mu }\sqrt{ \frac{(\mu +\gamma )(\mu +\upsilon )}{2}} \biggr) \rightarrow \Phi \biggl(\frac{\sigma \eta N}{\sqrt{2}} \biggr), \end{aligned}$$

thus, if $\eta \rightarrow 0$ (except for $\mu \rightarrow 0$), then $p \rightarrow 0$ since

$$\begin{aligned} \Phi \biggl( \sigma \eta N\frac{1}{\mu }\sqrt{ \frac{(\mu +\gamma )(\mu +\upsilon )}{2}} \biggr) \rightarrow \Phi (0) = 1/2. \end{aligned}$$

Analogously, for the SIS model with random perturbation the following holds:

$$\begin{aligned} P \biggl( R_{0,E}^{\mathrm{SIS}}\leq R_{0,v}^{\mathrm{SIS}}\leq \beta \frac{\eta N}{ \mu (\gamma +\mu )} + \frac{ \sigma ^{2}\eta ^{2}N^{2}}{2\mu ^{2} (\gamma +\mu )} \biggr) \rightarrow 1 \quad \text{if } \mu \rightarrow 0 \end{aligned}$$

and

$$\begin{aligned} P \biggl( R_{0,E}^{\mathrm{SIS}}\leq R_{0,v}^{\mathrm{SIS}}\leq \beta \frac{\eta N}{ \mu (\gamma +\mu )} + \frac{ \sigma ^{2}\eta ^{2}N^{2}}{2\mu ^{2} (\gamma +\mu )} \biggr) \rightarrow 0\quad \text{if } \eta \rightarrow 0 \text{ and } \mu \text{ does not tend to $0$}. \end{aligned}$$

We now discuss the confidence intervals and hypothesis tests from the basic reproduction. Let $R_{1},\ldots,R_{n}$ be the average number of cases of infected people for $1,\ldots,n$, respectively. According to the previously mentioned, we assume that $R_{1},\ldots,R_{n}\sim N ( R_{0}^{\mathrm{SIR}}, ( \eta ^{2} \upsilon ^{2}\sigma ^{2}N^{2} ) / ( 2\mu ^{2} ( \mu +\upsilon ) ^{3} ( \mu +\gamma ) ^{3} ) ) $, all independent. Note that

$$\begin{aligned} \bar{R}= \frac{R_{1}+\cdots +R_{n}}{n}\sim N \biggl( R_{0}^{\mathrm{SIR}}, \frac{\eta ^{2}\upsilon ^{2}\sigma ^{2}N^{2}}{2n\mu ^{2} ( \mu +\upsilon ) ^{3} ( \mu +\gamma ) ^{3}} \biggr), \end{aligned}$$

to determinate a confidence set under a confidence level $1-\alpha $, knowing *μ*, *β*, *γ*, *υ*, and *σ*, observe that

3.14 $$\begin{aligned} Z_{\alpha /2}< Z = \frac{\bar{R}-R_{0}^{\mathrm{SIR}}}{\frac{R^{\mathrm{SIR}}\sigma }{\sqrt{2n}\beta \sqrt{ ( \mu +\upsilon ) ( \mu +\gamma ) }}}< Z_{1-\alpha /2} \text{,} \end{aligned}$$

therefore,

$$\begin{aligned} Z_{\alpha /2} \frac{R_{0}^{\mathrm{SIR}}\sigma }{\sqrt{2n}\beta \sqrt{ ( \mu +\upsilon ) ( \mu +\gamma ) }}< \frac{\bar{R}-R_{0}^{\mathrm{SIR}}}{R_{0}^{\mathrm{SIR}}}< Z_{1-\alpha /2} \frac{R_{0}^{\mathrm{SIR}}\sigma }{\sqrt{2n}\beta \sqrt{ ( \mu +\upsilon ) ( \mu +\gamma ) }}. \end{aligned}$$

Thus,

$$\begin{aligned} Z_{\alpha /2} \frac{\sigma }{\sqrt{2n}\beta \sqrt{ ( \mu +\upsilon ) ( \mu +\gamma ) }}+1< \frac{\bar{R}}{R_{0}^{\mathrm{SIR}}}< Z_{1-\alpha /2} \frac{\sigma }{\sqrt{2n}\beta \sqrt{ ( \mu +\upsilon ) ( \mu +\gamma ) }}+1. \end{aligned}$$

Then

$$\begin{aligned} \frac{\sigma Z_{\alpha /2}+\bar{R}\sqrt{2n}\beta \sqrt{ ( \mu +\upsilon ) ( \mu +\gamma ) }}{\bar{R}\sqrt{2n}\beta \sqrt{ ( \mu +\upsilon ) ( \mu +\gamma ) }}< \frac{945}{R_{0}^{\mathrm{SIR}}}< \frac{\sigma Z_{1-\alpha /2}+\bar{R}\sqrt{2n}\beta \sqrt{ ( \mu +\upsilon ) ( \mu +\gamma ) }}{\bar{R}\sqrt{2n}\beta \sqrt{ ( \mu +\upsilon ) ( \mu +\gamma ) }}. \end{aligned}$$

Similarly, the confidence set is given by $( \bar{R} \frac{\beta \sqrt{2na}}{\sigma Z_{1-\alpha /2}+\bar{R}\sqrt{2na}\beta },\bar{R} \frac{\beta \sqrt{2na}}{\sigma Z_{\alpha /2}+\bar{R}\sqrt{2na}\beta } ) $, where $a= ( \mu +\upsilon ) ( \mu +\gamma ) $. For calculating the size of sample with an error *e*, see that

$$\begin{aligned} e=2Z_{1-\alpha /2} \frac{\sigma }{\sqrt{2n}\beta \sqrt{a}}, \end{aligned}$$

therefore,

$$\begin{aligned} n= \frac{2\sigma ^{2} ( Z_{1-\alpha /2} ) ^{2}}{e\beta ^{2} ( \mu +\upsilon ) ( \mu +\gamma ) }. \end{aligned}$$

The statistic test *Z* is given by (3.14) and the critical sets are $( Z_{1-\alpha }, + \infty )$, $(-\infty, Z_{1-\alpha } )$, and $(-\infty, - Z_{1-\alpha /2} ) \cup (Z_{1-\alpha /2} , +\infty ) $ for the alternative test $H_{0}: R^{\mathrm{SIR}}_{0} < r$, $H_{0}: R^{\mathrm{SIR}}_{0} > r$, and $H_{0}: R^{\mathrm{SIR}}_{0} \neq r$.

## Basic reproduction variable with double stochastic component

In this section, we determine the basic reproduction variable for the model based on the stochastic differential equations with two kinds of perturbation terms. We consider the *SEIRS epidemic model with stochastic transmission* proposed by Witbooi [26] to include two stochastic perturbation terms in the disease model. It is given by

4.1 $$\begin{aligned} \textstyle\begin{cases} dS(t)= ( \eta N -\beta I(t)S(t)+ \alpha R(t) - \mu S ( t ) ) \,dt-\sigma (p S(t) E(t) + q S(t) I(t) )\,dB ( t ) , \\ dE(t)= ( \beta I(t) S(t)-\upsilon E ( t ) -\mu _{1} E ( t ) ) \,dt+\sigma p S(t) E(t)\,dB ( t ) , \\ dI(t)= ( \upsilon E ( t ) -\gamma I(t)-\mu _{2} I ( t ) ) \,dt +\sigma q S(t) I(t)\,dB ( t ) ,\\ dR(t)= ( \gamma I(t) -\alpha R(t) - \mu _{3} R ( t ) ) \,dt.\end{cases}\displaystyle \end{aligned}$$

Analogously, the deterministic version of the SEIR model with demography is given by

4.2 $$\begin{aligned} \textstyle\begin{cases} dS(t)= ( \eta N -\delta S(t)E(t) -\beta S(t)I(t) -\xi S(t)I(t) + \alpha R(t) - \mu S ( t ) ) \,dt, \\ dE(t)= ( \beta I(t) S(t) + \delta S(t)E(t) - \upsilon E ( t ) -\mu _{1} E ( t ) ) \,dt, \\ dI(t)= ( \upsilon E ( t ) -\xi S(t)I(t) -\gamma I(t)-\mu _{2} I ( t ) ) \,dt ,\\ dR(t)= ( \gamma I(t) -\alpha R(t) - \mu _{3} R ( t ) ) \,dt. \end{cases}\displaystyle \end{aligned}$$

Using the approach of *the next generation matrix method*(see [5]) for the deterministic model, the matrix **T** (*transmissions*) and the matrix Σ (*transitions*), respectively, are given by

$$\begin{aligned} \mathbf{T} = \begin{pmatrix} \frac{\eta }{\mu } \delta N & \frac{\eta }{\mu } \beta N \\ 0 & \frac{\eta }{\mu } \xi N \end{pmatrix} \quad\text{and}\quad \Sigma = \begin{pmatrix} -(\upsilon + \mu ) & 0 \\ \upsilon & -(\gamma + \mu ) \end{pmatrix}, \end{aligned}$$

and

$$\begin{aligned} - \mathbf{T} \Sigma ^{-1} = \begin{pmatrix} \frac{\eta }{\mu } \delta N & \frac{\eta }{\mu } \beta N \\ 0 & \frac{\eta }{\mu } \xi N \end{pmatrix} \begin{pmatrix} \frac{1}{(\upsilon + \mu )} & 0 \\ \frac{\upsilon }{(\upsilon + \mu )(\gamma + \mu )} & \frac{1}{(\gamma + \mu )} \end{pmatrix}= \begin{pmatrix} \frac{\eta }{\mu } \frac{\delta N}{(\upsilon + \mu )} + \frac{\eta }{\mu } \frac{\upsilon \beta N}{(\upsilon + \mu )(\gamma + \mu )} & \frac{\eta }{\mu } \frac{\beta N}{(\gamma + \mu )} \\ \frac{\eta }{\mu } \frac{\upsilon \xi N}{(\upsilon + \mu )(\gamma + \mu )} & \frac{\eta }{\mu } \frac{\xi N}{(\gamma + \mu )} \end{pmatrix}. \end{aligned}$$

The eigenvalues of $- \mathbf{T} \Sigma ^{-1}$ correspond to

$$\begin{aligned} \lambda _{1,2} = \frac{{ \frac{\eta }{\mu } N (\beta \upsilon +\delta (\gamma + \mu ) + \xi (\mu + \upsilon ) ) \mp \frac{\eta }{\mu } N \triangle ^{1/2} }}{{ 2 (\gamma + \mu ) (\mu + \upsilon )}} \end{aligned}$$

with $\triangle = (\delta (\gamma + \mu ) + \beta \upsilon +\xi (\mu + \upsilon ) )^{2} -4 \xi \delta (\gamma + \mu ) (\mu + \upsilon )$. It is clear that the greatest eigenvalue is $\lambda _{2}$, which is the basic reproduction number for system (4.2).

For system (4.2), we assume that $F(a) = e^{-(\mu + \upsilon )(\mu + \gamma )a}$, and as in example (A.1) with function

$$\begin{aligned} b(a) = \frac{{\eta }}{{ 2 \mu }} N \bigl[ \beta \upsilon +\delta (\gamma + \mu ) + \xi (\mu + \upsilon ) + \triangle ^{1/2} \bigr]. \end{aligned}$$

**Figure 4:**
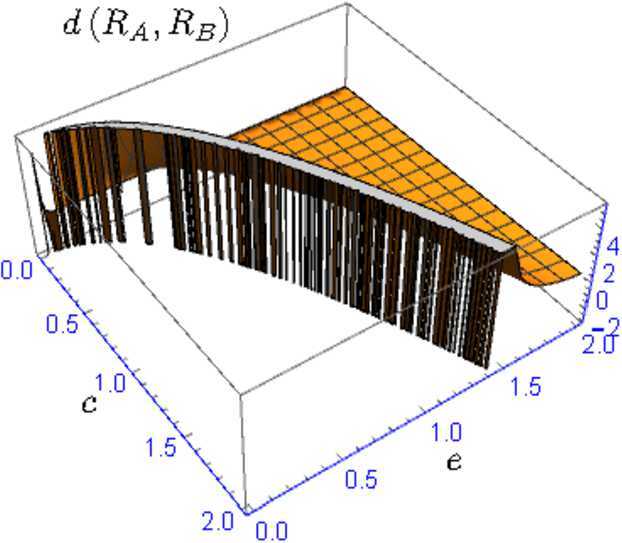
Graphic of the function $d(R_{A}, R_{B}) = k \times \frac{\sqrt{e}}{c^{2}-e}$ with $k = 1$ restricted to $\{ e,c: c^{2} - e \geq 0 \} $. Our case considers $k = \frac{2b}{(\mu + \upsilon )(\mu + \gamma )}$, a function which has similar behavior to $k \times \frac{\sqrt{e}}{c^{2}-e}$
  On the other hand, note that if $e \rightarrow 0$, then $d(R_{A}, R_{B}) \rightarrow 0$, which lets us conclude that $E(R) \rightarrow E ( R_{A} ) = E ( R_{B} )$. This happens when $\sigma \rightarrow 0$, $p \rightarrow 0$ or $q \rightarrow 0$. However, if $\sigma \rightarrow 0$ then $c \rightarrow 0$, thus $E ( R_{A} ) \rightarrow + \infty $. If $p \rightarrow 0$ and $q \rightarrow 0$ (at the same time), then $c \rightarrow 0$, thus $E ( R_{A} ) \rightarrow + \infty $. In case that $E ( R_{A} ) \rightarrow + \infty $, then $E ( R ) \rightarrow + \infty $, therefore the mean of $R_{0,v}^{\mathrm{SEIRS}}$ does not have sense.

For system (4.1), take $d \delta =\sigma p dB(t)$ and $d \xi = \sigma q dB(t)$ ([8] and [9]). Based on the construction of integral (3.6), we define the basic reproduction variable for the system:

4.3 $$\begin{aligned} R_{0,v}^{\mathrm{SEIRS}} = \frac{{\eta }}{{2 \mu }} N \int_{0}^{{+\infty } } \bigl[ \bigl( \beta \upsilon +\sigma \bigl( p (\gamma + \mu ) + q (\mu + \upsilon ) \bigr) B(a) + \sqrt{\triangle _{b}} \bigr) e^{-(\mu + \upsilon )( \mu + \gamma )a} \bigr] \,da, \end{aligned}$$

where $\triangle _{b}= (\sigma [ p (\gamma + \mu ) + q (\mu + \upsilon ) ] B(a) + \beta \upsilon )^{2} -4 pq \sigma ^{2} (\gamma + \mu ) (\mu + \upsilon ) B^{2}(a)$. Observe that

(i) $\int_{0}^{{+\infty } } \frac{{\eta \upsilon \beta N }}{{ 2 \mu }} e^{-(\mu + \upsilon )(\mu + \gamma ) a}\,da = \frac{\eta \upsilon \beta N}{2 \mu ( \mu +\upsilon ) ( \mu +\gamma )}$
(ii) $\int_{0}^{{+\infty } } s B(a) e^{-(\mu + \upsilon )( \mu + \gamma ) a}\,da \sim \mathbf{N} ( 0, \frac{ s^{2} }{2(\mu + \upsilon )^{3} (\mu + \gamma )^{3} } )$, with $s=\frac{\eta N}{2 \mu } \sigma [ p (\gamma + \mu ) + q (\mu + \upsilon ) ]$
(iii) Note that $\triangle _{b} (x ) = (c x - b )^{2} - e x^{2} = (c^{2}-e ) x^{2} + 2bc x + b^{2}$; where $b = \beta \upsilon $, $c = \sigma [ p (\gamma + \mu ) + q (\mu + \upsilon ) ]$ and $e = 4 pq \sigma ^{2} (\gamma + \mu ) (\mu + \upsilon )$. The roots of $\triangle _{b} (x )$ are given by
  $$\begin{aligned} x = \frac{{ -2bc \mp \sqrt{4b^{2}c^{2}-4(c^{2}-e)b^{2}} }}{{ 2(c^{2}-e) }} = \frac{{-2bc \mp \sqrt{4b^{2}e} }}{{ 2(c^{2}-e) }} = \frac{{ -bc \pm b \sqrt{e} }}{{ c^{2}-e }} = \frac{{ -b }}{{c \mp \sqrt{e}}}, \end{aligned}$$
  therefore
  $$\begin{aligned} &\int_{0}^{{+\infty } } \sqrt{\triangle _{b} \bigl(B(a) \bigr)} e^{-(\mu + \upsilon )(\mu + \gamma )a} \,da\\ &\quad = \int_{0}^{{+\infty } } \biggl( \biggl( B(a) + \frac{{ b }}{{c - \sqrt{e}}} \biggr) \biggl( B(a) + \frac{{b }}{{c + \sqrt{e}}} \biggr) \biggr)^{1/2} e^{-(\mu + \upsilon )( \mu + \gamma )a} \,da . \end{aligned}$$
  It is easy to observe that, for all $\omega \in \Omega $,
  $$\begin{aligned} &\int_{0}^{{+\infty } } \biggl( B ( a ) + \frac{b}{c+\sqrt{e}} \biggr) e^{- \phi a}\,da \\ &\quad\leq \int_{0}^{{+\infty } } \sqrt{\triangle _{b} \bigl(B(a) \bigr)} e^{-(\mu + \upsilon )(\mu + \gamma )a} \,da \leq \int_{0}^{{+\infty } } \biggl( B ( a ) + \frac{b}{c-\sqrt{e}} \biggr) e^{-\phi a}\,da \end{aligned}$$
  with $\phi = - ( \mu +\upsilon ) ( \mu +\gamma )$. Note by equation (3.7) that
  $$\begin{aligned} &\int_{0}^{{+\infty } }B ( a ) e^{- ( \mu +\upsilon ) ( \mu +\gamma ) a}\,da\\ &\quad\sim \mathbf{N} \biggl( -\frac{\lim_{l\rightarrow +\infty } B ( l ) e^{- ( \mu +\upsilon ) ( \mu +\gamma ) l}}{ ( \mu +\upsilon ) ( \mu +\gamma )}, \int_{0}^{{+\infty } } \frac{e^{-2 ( \mu +\upsilon ) ( \mu +\gamma ) a}}{ ( \mu +\upsilon ) ^{2} ( \mu +\gamma ) ^{2}}\,da \biggr), \end{aligned}$$
  due to $\lim_{l\rightarrow +\infty } B ( l ) e^{- ( \mu + \upsilon ) ( \mu +\gamma ) l} = 0$ (reasoning similarly to inequality (3.8)), note that $\int ^{+ \infty }_{0} ( B ( a ) + \frac{b}{c-\sqrt{e}} ) e^{- ( \mu +\upsilon ) ( \mu +\gamma ) a}\,da \sim \mathbf{N} ( 0,1 / ( 2 ( \mu +\upsilon ) ^{3} ( \mu +\gamma ) ^{3} ) )$.
  On the other hand, $\int_{0}^{{+\infty } }\frac{b}{c-\sqrt{e}} e^{- ( \mu +\upsilon ) ( \mu +\gamma ) a}\,da= \frac{b}{ ( c-\sqrt{e} ) ( \mu +\upsilon ) ( \mu +\gamma )}$, therefore $\int_{0}^{{+\infty } } ( B ( a ) + \frac{b}{c-\sqrt{e}} ) e^{- ( \mu +\upsilon ) ( \mu +\gamma ) a}\,da\sim \mathbf{N} ( \frac{b}{ ( c-\sqrt{e} ) ( \mu +\upsilon ) ( \mu +\gamma )}, \frac{1 }{2 ( \mu +\upsilon ) ^{3} ( \mu +\gamma ) ^{3}} ) $.
  Taking the random variables
  $$\begin{aligned} &R_{A}= \int_{0}^{{+\infty } } \biggl( B ( a ) +\frac{b}{c+\sqrt{e}} \biggr) e^{- ( \mu +\upsilon ) ( \mu +\gamma ) a}\,da \quad\text{and}\\ & R_{B}= \int_{0}^{{+\infty } } \biggl( B ( a ) +\frac{b}{c-\sqrt{e}} \biggr) e^{- ( \mu +\upsilon ) ( \mu +\gamma ) a}\,da, \end{aligned}$$
  we have $R_{A}$ and $R_{B}$ are normally distributed with variance $1 / ( 2 ( \mu +\upsilon ) ^{3} ( \mu + \gamma ) ^{3} ) $ and means $b / ( ( c+\sqrt{e} ) ( \mu +\upsilon ) ( \mu +\gamma ) ) $ and $b / ( ( c-\sqrt{e} ) ( \mu +\upsilon ) ( \mu +\gamma ) ) $, respectively. In addition, for all $\omega \in \Omega $, it is clear that
  $$\begin{aligned} R_{A}\leq \int_{0}^{{+\infty } } \biggl[ \biggl( B ( a ) + \frac{b}{c-\sqrt{e}} \biggr) \biggl( B ( a ) + \frac{b}{c+\sqrt{e}} \biggr) \biggr] ^{1/2}e^{- ( \mu +\upsilon ) ( \mu +\gamma ) a}\,da\leq R_{B}. \end{aligned}$$
  Writing $R= \int ^{+ \infty }_{0} [ ( B ( a ) + \frac{b}{c-\sqrt{e}} ) ( B ( a ) + \frac{b}{c+\sqrt{e}} ) ]^{1/2} e^{- ( \mu + \upsilon ) ( \mu +\gamma ) a} \,da$, we have that $E ( R_{A} ) \leq E ( R ) \leq E ( R_{B} )$. The distance between $E ( R_{A} )$ and $E ( R_{B} )$ corresponds to
  $$\begin{aligned} d ( R_{A}, R_{B} ) = \frac{2 b \sqrt{e} }{ ( c^{2}-e ) ( \mu +\upsilon ) ( \mu +\gamma )}. \end{aligned}$$
  Observe that
  $$\begin{aligned} c^{2} - e ={}& \sigma ^{2} \bigl[ p (\gamma + \mu ) + q (\mu + \upsilon ) \bigr]^{2} - 4 pq \sigma ^{2} (\gamma + \mu ) (\mu + \upsilon ) \\ ={}& \sigma ^{2} p^{2} (\gamma + \mu )^{2} + \sigma ^{2} 2 pq ( \gamma + \mu ) (\mu + \upsilon ) + \sigma ^{2} q^{2} (\mu + \upsilon )^{2} \\ &{}- 4 pq \sigma ^{2} (\gamma + \mu ) (\mu + \upsilon )\\ = {}&\sigma ^{2} \bigl( p(\gamma + \mu ) - q (\mu + \upsilon ) \bigr)^{2} \geq 0, \end{aligned}$$
  that is, $c^{2} - e \geq 0$. The Fig. 4 shows that the function $d ( R_{A}, R_{B} )$ is decreasing for all $c, e$ with $c^{2} - e \geq 0$. Therefore, when $e, c \rightarrow + \infty $, then $d(R_{A}, R_{B}) \rightarrow 0$, then $E(R) \rightarrow E ( R_{A} ) = E ( R_{B} )$. This happens when *σ*, *p*, *q*, *γ*, *μ*, or *υ* tends to ∞.

By the procedures done in items (i), (ii), and (iii) of this section, it is possible to see that the basic reproduction number of system (4.1), $R_{0,v}^{\mathrm{SEIRS}}$, is a random variable whose expectation holds

4.4 $$\begin{aligned} \frac{}{ ( \mu +\upsilon ) ( \mu +\gamma )} \biggl( \frac{\eta N}{2 \mu } + \frac{1 }{\sigma g } \biggr) \leq E \bigl( R_{0,v}^{\mathrm{SEIRS}} \bigr) \leq \frac{}{ ( \mu +\upsilon ) ( \mu +\gamma )} \biggl( \frac{\eta N}{2 \mu } + \frac{1 }{\sigma l } \biggr) \end{aligned}$$

with $g = [ \sqrt{p (\gamma + \mu )} + \sqrt{q (\mu + \upsilon )} ]^{2} $ and $l = [ \sqrt{p (\gamma + \mu )} - \sqrt{q (\mu + \upsilon )} ]^{2}$. If $q \rightarrow 0$, then $E ( R_{0,v}^{\mathrm{SEIRS}} ) = \frac{\upsilon \beta }{ ( \mu +\upsilon ) ( \mu +\gamma )} ( \frac{ \eta N}{2 \mu } + \frac{ 1}{\sigma g} )$.

## Conclusions

In this paper, we have studied the basic reproduction number in stochastic epidemic models to include random perturbations in the infection rate as the contributing factor for the spread of the epidemics. We have established stability conditions for the SIS, SIR, and SEIR epidemic models. As in the case of the deterministic SEIR model, the condition $R^{\mathrm{SEIR}}_{0} < 1$ is not enough for the disease-free equilibrium point to be asymptotically stable. We showed that it is also necessary that $\mu + \upsilon < 1$. Also, in some deterministic models, the basic reproduction number is defined as the survival probability, which coincides with the value $R_{0}$. If $R_{0}<1$, then the disease-free equilibrium point is asymptotically stable. However, epidemic models with random perturbations need not be the same. In this paper, we considered the basic reproduction number as a random variable. Under stability conditions (Theorems 2.3 and 2.2), we proved that the basic reproduction number depends on the perturbation parameter *σ*, which means that the variations can affect the epidemic spread. We also presented simulation results that the value of $R_{0}$ for which the disease-free equilibrium point is asymptotically stable is less than the value found in the proofs of Theorems 2.3 and 2.2. Finally, we presented conjectures (3.1) and (3.2) to conclude that the transmission velocity of an epidemic is lower than the variation fluctuations, and for the values of $R_{0}$ proved in Theorems 2.3 and 2.2. The limitation of the proposed model is that populations that make transitions to the compartment are assumed to interact homogeneously and death rates are equal. The future work in this direction comprises considering a more realistic scenario using data from the recent COVID-19 outbreak in the city of Bogotá to include the lockdown restrictions and social mobility in the spread of infections that would allow us to address the issue of dependence control measures and epidemics mitigation.

## Data Availability

Not applicable. All data generated or analysed during this study are simulated and included in this manuscript.

## Appendix A: Construction of the basic reproduction number on deterministic epidemic models

### Example A.1

(Basic reproduction number in a deterministic SEIR model with demography)

Let be $P ( a ) =$“number of exposed population which become infected individuals and remain infected from the time 0 to *a*”. Note that the number of individuals per unit of time which avoid being exposed people during the period $[ 0,a ]$ are those that died or who became infected individuals, that is, $( \mu +\upsilon ) P ( a ) $ individuals per unit of time. The individuals who recovered from the disease or died are those who do not remain infected during the period $[ 0,a ]$, namely $( \mu +\upsilon ) ( \mu +\gamma ) P ( a ) $ individuals per unit of time. The others continue being exposed people or they are infected individuals which remain infected during $[ 0,a ] $.

$$\begin{aligned} \frac{\,dP(a)}{da}=- ( \mu +\upsilon ) ( \mu +\gamma ) P ( a ), \end{aligned}$$

solving the differential equation, we get

$$\begin{aligned} P ( a ) =P ( 0 ) e^{- ( \mu +\upsilon ) ( \mu +\gamma ) a}. \end{aligned}$$

Initially it needs to have at least an infected individual or an exposed individual which becomes infected, for when the epidemic occurs, then $P ( 0 ) $ is the number of initial infected people. $P ( a ) $ is the number of infected individuals which remain infected during the period $[0,a ] $. Note that $P ( a ) $ corresponds to $P(0)$ multiplied by the probability that an infected individual continues to be infected during all the interval $[0,a ] $. Therefore, $e^{- ( \mu +\upsilon ) ( \mu +\gamma ) a}$ the probability previously described. In this way,

$$\begin{aligned} F ( a ) =e^{- ( \mu +\upsilon ) ( \mu + \gamma ) a} \end{aligned}$$

is the survival function.

On the other hand, if an infected individual, $I (0 )=1$, arrives at a place where the population is completely susceptible, $S (0 ) =N$, then it is expected to have *βN* exposed individuals in total. From the *βN* expected exposed individuals, $\upsilon \beta N$ corresponds to the total infected population, therefore

$$\begin{aligned} b ( a ) =\upsilon \beta N \end{aligned}$$

as long as the mortality rate is the same as the birth rate. In another case, note that

$$\begin{aligned} \frac{dN(t) }{dt } = \eta N - \mu \bigl( S(t) + E(t) + I(t) + R(t) \bigr) = \eta N - \mu \bigl( N(t) \bigr), \end{aligned}$$

and the solution of the above equation is given by

$$\begin{aligned} N(t) &= S ( t ) +E ( t ) +I ( t ) +R ( t ) = \biggl[N ( 0 ) + \int ^{t}_{0} e^{\mu v} \eta N \,dv \biggr] e^{-\mu t} \\ &= N ( 0 ) e^{-\mu t} + \frac{\eta }{\mu } N - \frac{\eta }{\mu } N e^{-\mu t}, \end{aligned}$$

when $t \to \infty $, then $N(t)$ tends to $\frac{\eta }{\mu } N$. Therefore, if an infected individual arrives in a completely susceptible population, then it will have $\frac{\eta }{\mu } N$ new infected on an enough big period of time. Thus, the function $b ( a )$ is given by

$$\begin{aligned} b ( a ) = \frac{\eta }{\mu }\upsilon \beta N, \end{aligned}$$

then the basic reproduction number for the model SEIR with demography is

A.1 $$\begin{aligned} R_{0}^{\mathrm{SEIR}}:= \int_{0}^{{+\infty } } \frac{\eta }{\mu }\upsilon \beta Ne^{- ( \mu +\upsilon ) ( \mu + \gamma ) a}\,da= \frac{\eta }{\mu } \frac{\upsilon \beta N}{ ( \mu +\upsilon ) ( \mu +\gamma )}. \end{aligned}$$

## Appendix B: Stability on deterministic epidemic models

We give the following theorem which gives sufficient conditions for a point to be asymptotically stable using the appropriate Lyapunov functions ([12] and [25]).

### Theorem B.1

*Let* $X ( 0 ) $ *be an equilibrium point of system* (2.6) (*in the other case*, *it is possible to do the substitution* $\xi (t)=X(t)-\overset{\circ }{x}$, *where* $\overset{\circ }{x}$ *is an equilibrium point*) *defined for all* $t\geq 0$, *and* $V:\mathbb{R}^{n}\rightarrow \mathbb{R}$ *is a Lyapunov function*. *Then*, *for some* $t_{0}$,

(*i*) *If* *V* *satisfies that*

B.1 $$\begin{aligned} \dot{V} \bigl( X ( t ) \bigr) \leq 0\quad\textit{for all }t \geq t_{0}, \end{aligned}$$

*then* $X ( 0 ) $ *is stable*.

(*ii*) *If* *V* *satisfies* (i) *and furthermore*

B.2 $$\begin{aligned} \dot{V} \bigl( X ( t ) \bigr) < 0\quad\textit{for all }t\geq t_{0}, \end{aligned}$$

*then* $X ( 0 ) $ *is asymptotically stable*.

To prove the *stability* of an ordinary equation system, we use construction of the *Lyapunov functions*. The definition is given in the following definition given in [12].

### Definition B.1

Let $\dot{X} ( t ) =f (X ( t ) ) $ be an ordinary differential equation system defined for all $t\geq 0$, and let $V: \mathbb{R}^{n} \rightarrow \mathbb{R}$ be a continuous function with continuous derivatives.

(i) The *rate of* *V* *with respect to* $X_{1} ( t ),\ldots,X_{n} (t ) $ is defined as

B.3 $$\begin{aligned} \dot{V} \bigl( X ( t ) \bigr):= \frac{dV ( X ( t ) )}{dt} = \sum_{i=1}^{{n} } \frac{\partial V}{\partial X_{i}} \frac{dX_{i} ( t )}{dt}. \end{aligned}$$

(ii) If *V* satisfies that $V ( X ( 0 ) ) =0$ and $V ( X ( t ) ) >0$ for all $t>0$, then *V* is called a *Lyapunov function*.

We give the theorems which relate with the basic reproduction number and disease-free equilibrium point for the deterministic models. The proofs are based on [18] and [27]. Now we give the following theorem for the SEIR model with the demography.

### Theorem B.2

*If* $R^{\mathrm{SEIR}}_{0} < 1$ *and* $\upsilon +\mu < 1$, *then* $( \frac{\eta }{\mu }N,0,0,0 )$ *is asymptotically stable in the SEIR model with demography*.

### Proof

Assume $\eta \geq \mu $. Define the function *W* given by

$$\begin{aligned} W \bigl( S ( t ),E ( t ),I ( t ),R ( t ) \bigr) =\lambda _{1} \biggl( \frac{\eta }{\mu }N-S \biggr) ^{2}+\lambda _{2} \biggl( \upsilon EI+\upsilon ^{2} \frac{1 }{2}E^{2}+ ( \mu + \upsilon ) \frac{1 }{2}I^{2} \biggr) + \frac{1 }{2} \lambda _{3}R^{2}, \end{aligned}$$

where $\lambda _{1},\lambda _{2},\lambda _{3}>0$ are positive constants adequately chosen. Clearly $W ( S ( t ),E ( t ),I ( t ), R ( t ) ) >0$ for all $t>0$ and $W ( N,0,0,0 ) =0$. Given $S ( t ) $, $E ( t ) $, $I ( t ) $, and $R ( t ) $ are continuous functions and

$$\begin{aligned} &\frac{\partial W}{\partial S ( t )}=-2\lambda _{1} \biggl( \frac{\eta }{\mu }N-S \biggr),\qquad \frac{\partial W}{\partial E ( t )}=\lambda _{2} \bigl( \upsilon ^{2}E+\upsilon I \bigr), \\ & \frac{\partial W}{\partial I ( t )}=\lambda _{2} \bigl( \upsilon E+ ( \mu +\upsilon ) I \bigr) y \frac{\partial W}{\partial R ( t )}= \lambda _{4}R \end{aligned}$$

are continuous too, then *V* is a Lyapunov function. Notice that

$$\begin{aligned} \dot{W} \bigl( X ( t ) \bigr) ={}& \begin{pmatrix} \frac{\partial W}{\partial S ( t )}, & \frac{\partial W}{\partial E ( t )}, & \frac{\partial W}{\partial I ( t )}, & \frac{\partial W}{\partial R ( t )}\end{pmatrix} \frac{dX ( t )}{dt} \\ ={}& \begin{pmatrix} -2\lambda _{1} ( \frac{\eta }{\mu }N-S ) \\ \lambda _{2} ( \upsilon ^{2}E+\upsilon I ) \\ \lambda _{2} ( \upsilon E+ ( \mu +\upsilon ) I ) \\ \lambda _{4}R\end{pmatrix} ^{T} \begin{pmatrix} \eta N-\beta IS-\mu S \\ \beta IS-\upsilon E-\mu E \\ \upsilon E-\gamma I-\mu I \\ \gamma I-\mu R\end{pmatrix} \\ ={}&{ -}2\lambda _{1} \biggl( \frac{\eta }{\mu }N-S \biggr) ( \eta N- \beta IS-\mu S ) +\lambda _{2} \bigl( \upsilon ^{2}E+ \upsilon I \bigr) ( \beta IS-\upsilon E-\mu E ) \\ &{}+\lambda _{2} \bigl( \upsilon E+ ( \mu +\upsilon ) I \bigr) ( \upsilon E-\gamma I-\mu I ) +\lambda _{3} R ( \gamma I-\mu R ) \\ ={}&{ -}2\lambda _{1} \biggl( \frac{\eta }{\mu }N-S \biggr) ( \eta N- \beta IS-\mu S ) +\lambda _{2} \bigl( \upsilon ^{2}\beta SEI- \upsilon ^{2} ( \upsilon +\mu ) E^{2} \\ &{}+ \upsilon \beta SI^{2}-\upsilon ( \upsilon +\mu ) EI+\upsilon ^{2}E^{2}-\upsilon ( \gamma +\mu ) EI+\upsilon ( \mu + \upsilon ) EI \\ &{}- ( \gamma +\mu ) ( \mu +\upsilon ) I^{2} \bigr) +\lambda _{3} \bigl( \gamma RI-\mu R^{2} \bigr) \\ \leq{}& 2\lambda _{1}a ( t ) +\lambda _{2}b ( t ) +\lambda _{3}c ( t ), \end{aligned}$$

where^3^

$$\begin{aligned} &a(t)= \biggl( \frac{\eta }{\mu }N-S \biggr) ( -\eta N+\beta IS+\mu S ), \\ &b(t) = \bigl[ \bigl( \upsilon ^{2}\beta S -\upsilon ( \gamma +\mu ) \bigr) + \bigl( \upsilon ^{2}-\upsilon ^{2} ( \upsilon +\mu ) \bigr) + \bigl( \upsilon \beta S - ( \gamma +\mu ) ( \mu +\upsilon ) \bigr) \bigr]\\ &\phantom{b(t) =}{}\times \inf_{t\geq 0}\bigl\{ EI, E^{2}, I^{2} \bigr\} , \\ &c(t)=\gamma RI-\mu R^{2}. \end{aligned}$$

Now, it is an objective to show that $b(t) < 0$ for all $t \geq t_{0}$ with $t_{0}>0$. If $\eta \geq \mu $

(i) As $\upsilon \beta S< \upsilon \beta \frac{\eta }{\mu }N< ( \gamma +\mu ) ( \mu +\upsilon ) $ for all $t \geq 0 $ (by $R^{\mathrm{SEIR}}_{0}<1$), then
  $$\begin{aligned} &\upsilon [ \upsilon \beta S ] -\upsilon ( \gamma +\mu ) +\upsilon ^{2}-\upsilon ^{2} ( \upsilon +\mu ) \\ &\quad\leq \upsilon \biggl[ \upsilon \beta \frac{\eta }{\mu }N \biggr] -\upsilon ( \gamma +\mu ) +\upsilon ^{2}-\upsilon ^{2} ( \upsilon + \mu ) \\ &\quad< \upsilon \bigl[ ( \gamma +\mu ) ( \mu +\upsilon ) \bigr] - \upsilon ( \gamma +\mu ) +\upsilon ^{2}-\upsilon ^{2} ( \upsilon + \mu ) \\ &\quad= \bigl[ \upsilon ( \gamma +\mu ) -\upsilon ^{2} ( \upsilon + \mu ) \bigr] [ \upsilon +\mu -1 ] \\ &\quad< \bigl[ \upsilon ( \gamma +\mu ) \bigr] [ \upsilon +\mu -1 ] \end{aligned}$$
  when $\upsilon +\mu < 1$ then $\upsilon ^{2}\beta N-\upsilon ( \gamma +\mu ) + \upsilon ^{2}-\upsilon ^{2} ( \upsilon +\mu ) <0$.
(ii) Notice that $\upsilon \beta S< \upsilon \beta \frac{\eta }{\mu }N $ for all $t> 0$ and as
  $$\begin{aligned} \frac{\eta }{\mu } \frac{\upsilon \beta N}{ ( \mu +\upsilon ) ( \mu +\gamma )}=R_{0}^{\mathrm{SEIR}}< 1, \end{aligned}$$
  then $\upsilon \beta S - ( \mu +\gamma ) ( \mu + \upsilon ) \leq \upsilon \beta \frac{\eta }{\mu }N - ( \mu +\gamma ) ( \mu +\upsilon ) <0$, therefore
  $$\begin{aligned} \bigl( \upsilon \beta S- ( \mu +\gamma ) ( \mu + \upsilon ) \bigr) I^{2} ( t ) < 0, \end{aligned}$$

so by (i) and (ii) we have that $\lambda _{2}b ( t ) <0 $. In this way it is possible to chose the values for $\lambda _{1},\lambda _{2},\lambda _{3}$ that hold on

$$\begin{aligned} \dot{W} ( t ) \leq 2\lambda _{1}a ( t ) + \lambda _{2}b ( t ) +\lambda _{4}c ( t ) < 0. \end{aligned}$$

Then by Theorem B.1 it is concluded that $( \frac{\eta }{\mu }N,0,0,0 ) $ for the SEIR model with demography is asymptotically stable.

If $\mu >\eta $, and also for all $t>0$ for which $S(t) \leq \frac{\eta }{\mu }N$, it is clear that $\beta S(t) \leq \beta \frac{\eta }{\mu }N $. If there exists $t>0$ for which $S(t) > \frac{\eta }{\mu }N$, analogously like it was made for the proof of Theorem B.3 and following that

$$\begin{aligned} \frac{dS(t)}{dt}=\eta N-\beta I(t)S(t)-\mu S ( t ) < 0, \end{aligned}$$

it is shown that $\upsilon [ \upsilon \beta S ] -\upsilon ( \gamma + \mu ) +\upsilon ^{2}-\upsilon ^{2} ( \upsilon +\mu ) <0 $ and $\upsilon \beta S - ( \mu +\gamma ) ( \mu + \upsilon )<0$ for all $t > t_{0}$ for some $t_{0}>0$.

For any case, we have that $b(t)<0$, which is why it is possible to choose adequate values for $\lambda _{1}$, $\lambda _{2}$, and $\lambda _{3}$ such that

$$\begin{aligned} \dot{W} ( t ) \leq 2\lambda _{1}a ( t ) + \lambda _{2}b ( t ) +\lambda _{3}c ( t ) < 0. \end{aligned}$$

In consequence, the point $( \frac{\eta }{\mu }N,0,0,0 )$ is asymptotically stable in the SEIR model with demography. □

### Theorem B.3

*If* $R_{0}^{\mathrm{SIR}} < 1$ *and* $R_{0}^{\mathrm{SIS}} < 1$, *then* $( \frac{\eta }{\mu }N,0,0 ) $ *and* $( \frac{\eta }{\mu }N,0 ) $ *are asymptotically stable in* (*i*) *SIR y* (*ii*) *SIS models with demography*, *respectively*.

### Proof

The proof is similar to the previous theorem, taking *V* defined by

$$\begin{aligned} V \bigl( S ( t ),I ( t ),R ( t ) \bigr):=\lambda _{1} \biggl( \frac{\eta }{\mu }N-S ( t ) \biggr) ^{2}+\lambda _{2} \frac{1 }{2}I^{2} ( t ) +\lambda _{3} \frac{1428}{2}R^{2} ( t ), \end{aligned}$$

where $\lambda _{1},\lambda _{2},\lambda _{3}>0$ are appropriately chosen positive constants. □

## Footnotes

1 by the L’Hôpital’s rule.

2 by the L’Hôpital’s rule.

## References

[1] Bernt Ø. (2013). Stochastic Differential Equations: An Introduction with Applications.

[2] Beyer R.M., Manica A., Mora C. (2021). Shifts in global bat diversity suggest a possible role of climate change in the emergence of SARS-CoV-1 and SARS-CoV-2. Sci. Total Environ..

[3] Cai Y., Jiao J., Gui Z., Liu Y., Wang W. (2018). Environmental variability in a stochastic epidemic model. Appl. Math. Comput..

[4] Diekmann O., Heesterbeek J.A.P., Metz J.A. (1990). On the definition and the computation of the basic reproduction ratio $r_{0}$ in models for infectious diseases in heterogeneous populations. J. Math. Biol..

[5] Diekmann O., Heesterbeek J.A.P., Roberts M.G. (2009). The construction of next-generation matrices for compartmental epidemic models. J. R. Soc. Interface.

[6] Dietz K. (1993). The estimation of the basic reproduction number for infectious diseases. Stat. Methods Med. Res..

[7] Divine W. (2017). Complete global analysis of a two-scale network SIRS epidemic dynamic model with distributed delay and random perturbations. Appl. Math. Comput..

[8] El Fatini M., Laaribi A., Pettersson R., Taki R. (2019). Lévy noise perturbation for an epidemic model with impact of media coverage. Stochastics.

[9] Gray A., Greenhalgh D., Hu L., Mao X., Pan J. (2011). A stochastic differential equation SIS epidemic model. SIAM J. Appl. Math..

[10] Heesterbeek J.A.P. (2002). A brief history of $r_{0}$ and a recipe for its calculation. Acta Biotheor..

[11] Heffernan J.M., Smith R.J., Wahl L.M. (2005). Perspectives on the basic reproductive ratio. J. R. Soc. Interface.

[12] Hokayem, P.A., Gallestey, E.: Lyapunov Stability Theory. Nonlinear Syst. Control, Spring (2015)

[13] Ji C., Jiang D. (2017). The threshold of a non-autonomous SIRS epidemic model with stochastic perturbations. Math. Methods Appl. Sci..

[14] Kermack W.O., McKendrick A.G. (1927). Contribution to the mathematical theory of epidemics. Proceedings of the Royal Society of London. Series A, Containing Papers of a Mathematical and Physical Character.

[15] Khasminskii R. (2011). Stochastic Stability of Differential Equations.

[16] Klebaner F.C. (2012). Introduction to Stochastic Calculus with Applications.

[17] Lahrouz A., Omari L., Kiouach D. (2011). Global analysis of a deterministic and stochastic nonlinear SIRS epidemic model. Nonlinear Anal., Model. Control.

[18] Lahrouz A., Settati A. (2014). Necessary and sufficient condition for extinction and persistence of SIRS system with random perturbation. Appl. Math. Comput..

[19] Lai Chung K., Williams R.J., Williams R.J. (1990). Introduction to Stochastic Integration.

[20] Lin Y., Jiang D., Xia P. (2014). Long-time behavior of a stochastic SIR model. Appl. Math. Comput..

[21] Liu W., Mao X. (2013). Strong convergence of the stopped Euler–Maruyama method for nonlinear stochastic differential equations. Appl. Math. Comput..

[22] Mao X. (2008). Stochastic Differential Equations and Applications.

[23] Morris Q. (2010). Analysis of a co-epidemic model. SIAM Undergrad. Res. Online.

[24] Sturrock R.N., Frankel S.J., Brown A.V., Hennon P.E., Kliejunas J.T., Lewis K.J., Worrall J.J., Woods A.J. (2011). Climate change and forest diseases. Plant Pathol..

[25] Tornatore E., Buccellato S.M., Vetro P. (2005). Stability of a stochastic SIR system. Phys. A, Stat. Mech. Appl..

[26] Witbooi P.J. (2017). An SEIRS epidemic model with stochastic transmission. Adv. Differ. Equ..

[27] Yang Q., Jiang D., Shi N., Ji C. (2012). The ergodicity and extinction of stochastically perturbed SIR and SEIR epidemic models with saturated incidence. J. Math. Anal. Appl..

[28] Zhou Y., Zhang W., Yuan S. (2014). Survival and stationary distribution of a SIR epidemic model with stochastic perturbations. Appl. Math. Comput..

